# A novel lipidic peptide with potential to promote balanced effector-regulatory T cell responses

**DOI:** 10.1038/s41598-022-15455-5

**Published:** 2022-07-01

**Authors:** Michael Agrez, Justyna Rzepecka, Darryl Turner, Gavin Knox, Christopher Chandler, Christopher B. Howard, Nicholas Fletcher, Kristofer Thurecht, Stephen Parker, Hayley Gooding, Laura Gallagher

**Affiliations:** 1 InterK Peptide Therapeutics Limited, New South Wales, Australia; 2 Concept Life Sciences, Edinburgh, Scotland; 3 Auspep Pty Limited, Melbourne, Australia; 4 Centre for Advanced Imaging, University of Queensland, Brisbane, Australia; 5 Australian Institute for Bioengineering and Nanotechnology and the ARC Training Centre for Innovation in Biomedical Imaging Technologies, University of Queensland, Brisbane, Australia

**Keywords:** Dendritic cells, Regulatory T cells, T-helper 1 cells, Interferons, Interleukins, Autoimmunity, Signal transduction, Immunotherapy, CD8-positive T cells, Cytotoxic T cells, Cancer immunotherapy

## Abstract

T cell-dendritic cell (DC) interactions contribute to reciprocal stimulation leading to DC maturation that results in production of interleukin-12 (IL-12) and interferon-gamma (IFN-γ). Both cytokines have been implicated in autoimmune diseases while being necessary for effective immune responses against foreign antigens. We describe a lipidic peptide**,** designated IK14004, that modifies crosstalk between T cells and DCs resulting in suppression of IL-12p40/IFN-γ production. T cell production of interleukin-2 (IL-2) and IFN-γ is uncoupled and IL-12p70 production is enhanced. IK14004 induces expression of activating co-receptors in CD8+ T cells and increases the proportion of Foxp3-expressing CD4+ T regulatory cells. The potential for IK14004 to impact on signalling pathways required to achieve a balanced immune response upon stimulation of DCs and T cells is highlighted. This novel compound provides an opportunity to gain further insights into the complexity of T cell-DC interactions relevant to autoimmunity associated with malignancies and may have therapeutic benefit.

## Introduction

Autoimmune diseases and cancer are linked^1^ and cancer immunotherapy has highlighted this further by exacerbating autoimmune pathologies^2^. T effector (Teff) cells and immunosuppressive T regulatory (Treg) cells maintain immune homeostasis through mutual regulation and loss of homeostatic balance contributes to both autoimmunity and cancer^3^. Autoimmune and inflammatory conditions are also related to dysregulated activity by agonistic cytokines such as interleukin-12p40 (IL-12p40) and interferon-gamma (IFN-γ)^4–7^ that positively regulate one another^8,9^. Antigen-dependent T cell activation induced via the CD3-T cell receptor complex leads to interleukin-2 (IL-2) production which induces cell cycle progression and expression of the high-affinity IL-2 receptor subunit, CD25, on CD4+ and CD8+ T cells^10,11^. IL-2 is produced primarily by activated CD4+ T cells that drives T cell differentiation towards CD4+ Tregs^12^ and Tregs do not produce IL-2^13^. However, low levels of IL-2 are required to sustain immunosuppressive Foxp3-expressing CD4 + CD25 + Tregs via signalling through IL-2 receptor complex^14,15^. Excess Treg activity can lead to cancer whereas too little results in autoimmunity^11^. Given that the T cell co-receptor CD28 is expressed on both activated Teffs and Tregs^3^, redundancy between CD28- and IL-2-mediated homeostatic signals in Tregs cannot be excluded^11^. Manipulating IL-2 and its receptor can dramatically shift the balance between IL-2-producing effector T cells and IL-2-responsive Tregs^13^ and competing feedback loops regulated by expression levels of CD25 modulate IL-2 signalling between T helper cells and Tregs^16^. Hence**,** IL-2 promotes a variety of effector T cell responses that include CD8+ T cell-mediated cytotoxicity while being indispensable for the function of Tregs—the very cells that serve to suppress effector T cell responses^17^.

Under steady state conditions, potential autoreactive T cells are silenced by dendritic cells (DCs) and the induction of immunosuppressive Foxp3-expressing T regulatory (Treg) cells^18^. In response to pathogen- and cancer-related antigens, IL-2-responsive cells overcome the suppressive Treg phenotype^19,20^ and the ability of DCs to initiate an immune response is determined by their state of maturation^18^. Immature DCs require maturation signals to undergo functional changes that result in competent antigen-presenting capacity^20^. These signals include interactions between CD40 ligand (CD40L) expressed on CD4+ T cells and the CD40 receptor on DCs^21,22^ and interactions between co-receptors on T cells and co-stimulatory ligands on DCs^23–25^. CD40L-mediated licensing of DCs by CD4+ T cells is required to generate a robust CD8+ T cell response^26^.

IL-12 is also involved in DC maturation^27^ and production of IL-12 by mature DCs regulates IFN-γ production by pro-inflammatory Th1-differentiated T cells^21^. In contrast to the IL-12p70 heterodimer comprising p40/p35 subunits that promotes induction of Th1-like Tregs^28^, IL-12p40 suppresses Tregs^29^ and induction of IL-12p40 and p35 genes have different requirements for de novo protein synthesis^30^. For example, the interaction between CD40L and CD40 plays a critical role in IL-12p40 mRNA accumulation but not p35 mRNA^31^. Maturation of DCs varies with their IL-12-producing capacity although whether DC maturation can be judged solely on IL-12p40 production remains to be established^32^. Importantly, Tregs restrain IL-12-instigated Th1 pro-inflammatory responses that lead to IFN-γ production^33^.

The exact mechanisms underlying many of the interacting signalling pathways that involve kinases and transcriptional regulation of cytokine production which impact on immune responses remain to be defined. For example, the threshold of T cell activation is regulated by the Src family kinase (SFK) member, lymphocyte-specific protein tyrosine kinase (Lck)^34,35^ while the germinal centre kinase, HPK1 (MAPKIV), suppresses TCR-activation of the transcriptional regulator, activator protein 1 (AP-1) and extracellular-signal regulated kinase (ERK)^36^ associated with IL-2 transcription^37^. Interestingly, HPK1 acts to maintain the TCR activation threshold in T cells^38^ yet its catalytic activity is dependent on Lck^36^. Moreover, HPK1 is an important proximal mediator of nuclear factor kappa B (NF-kB) activation in T cells^39^ and NF-kB binds to the IL-12p40 promoter^40,41^. NF-kB negatively regulates the p35 subunit of IL-12p70 via a calcium-dependent mechanism in contrast to ERK signalling which mediates negative feedback on regulation of p40, but not p35^42^.

Activation of the IL-12p40 promoter is also induced by nuclear factor of activated T cells, NFAT, via the calcium-dependent calmodulin-calcineurin (CaM:CaN) pathway^43,44^ and NFAT is expressed in T cells and DCs^45^. Given the role of NFAT proteins in regulating transcription of IFN-γ^46,47^, suppression of NFAT attenuates IFN-γ-activated endogenous IL-12p40 mRNA expression^48^. In addition, IFN-γ production in DCs is dependent on a Janus kinase (JAK) family member, Tyk2, that signals via signal transducers and activators of transcription (STATs)^49^ and within DCs, c-Src contributes to calcium-dependent signalling^50^. Hence, targeting calcineurin-dependent NFAT signalling pathways and JAK/STATs have become attractive options against autoimmune diseases^51,52^. We have previously reported that an ERK-binding peptide, RSKAKNPLYR, inhibits c-Src activity^53^. Linkages between a peptide drug and a branched lipid unit are thought to enhance drug stability and generate a membrane-like structure with increased lipophilicity^54^. Moreover, the presence of long alkyl side chains serves to protect peptide moieties from enzymatic attack within cells^54^. Given the protective effects of hydrophobic dodecanoic (lauric) acid on intracellular protein degradation^55^, we sought to determine whether a lipidic peptide comprising RSKAKNPLYR linked to branched dodecanoic acid residues, designated IK14004, can modulate immune responses by peripheral blood mononuclear cells isolated from healthy volunteers.

## Results

### IK14004 enhances expression of IL-2 and CD25 in T cells in the absence of APCs.

**Figure 1:**
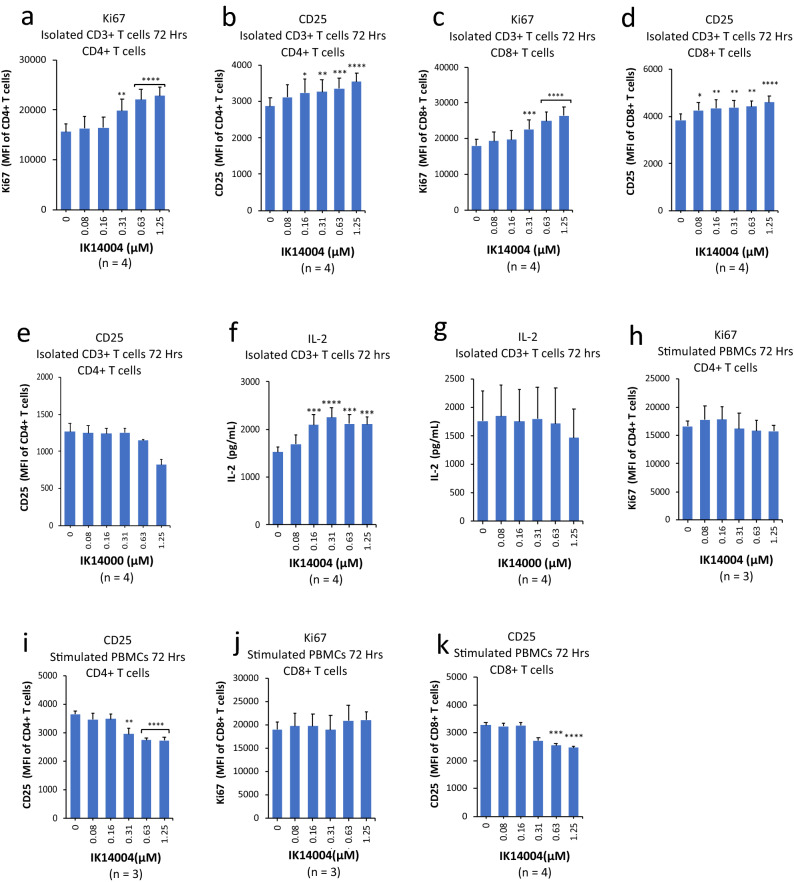
IK14004 enhances expression of IL-2 and CD25 in T cells in the absence of APCs. Buffy coat samples were obtained from human volunteers following ethics approval. PBMC and isolated CD3+ T cell cultures were stimulated with either anti-CD3 antibody alone or anti-CD3/anti-CD28 antibodies, respectively, as described in the “Methods”. Each tissue culture experiment was performed using triplicate wells (technical replicates) and cell-based flow cytometry/ELISA experiments were repeated at least three times (n = experimental replicates) as indicated below each panel. All error bars represent standard error of the mean (SEM). In all cell-based experiments the culture duration was 72 h. The compounds shown are IK14004 and IK14000, i.e., RSKAKNPLYR (IK14000) minus the dodecanoic acid moiety, as indicated under each panel. Flow cytometry data are shown as mean fluorescence intensity (MFI). Dot plots and gating strategies are shown in Supplementary Figs. S1 to S9. (**a**) Expression of Ki67 in CD4+ T cells within T cell cultures. (**b**) Expression of CD25 in CD4+ T cells within T cell cultures. (**c**) Expression of Ki67 in CD8+ T cells within T cell cultures. (**d**) Expression of CD25 in CD8+ T cells within T cell cultures. (**e**) Expression of CD25 in CD4+ T cells within T cell cultures exposed to peptide IK14000 lacking the dodecanoic acid moiety. The effect of dodecanoic acid residues alone could not be assessed in cell-based studies because of insolubility in physiological excipients. (**f**) IL-2 levels in supernatant from T cell cultures exposed to IK14004. (**g**) IL-2 levels in supernatant from T cell cultures exposed to IK14000. (**h**) Expression of Ki67 in CD4+ T cells within PBMC cultures exposed to IK14004. (**i**) Expression of CD25 in CD4+ T cells within PBMC cultures exposed to IK14004. (**j**) Expression of Ki67 in CD8+ T cells within PBMC cultures exposed to IK14004. (**k**) Expression of CD25 in CD8+ T cells within PBMC cultures exposed to IK14004.

Peripheral blood mononuclear cell (PBMC) and isolated CD3+ T cell cultures were stimulated with anti-CD3 and anti-CD3/anti-CD28 antibodies, respectively. We first sought to examine the effect of IK14004 on proliferative capacity and expression of the high-affinity IL-2 receptor-alpha subunit (IL-12Rα; CD25) in CD4+/CD8+ T cells together with IL-2 in supernatants from stimulated CD3+ T cells cultures after 72 h. Proliferative capacity as assessed by expression of Ki67 was enhanced in CD4+ T cells (Fig. 1a) and CD8+ T Cells (Fig. 1c). Similarly, CD25 expression increased in a dose-dependent manner in CD4+ T cells (Fig. 1b) and in CD8+ T cells (Fig. 1d). In contrast, in the absence of the dodecanoic acid moiety, i.e., the unconjugated 10-mer peptide, RSKAKNPLYR (IK14000), induced a decrease in CD25 expression in CD4+ T cells at higher concentrations (Fig. 1e). Expression of CD25 is regulated by IL-2^16^ and enhanced production of IL-2 was seen in the presence of IK14004 (Fig. 1f) but not in the presence of IK14000 which lacked the dodecanoic acid moiety (Fig. 1g). The dodecanoic moiety could not be assessed on its own because of insolubility in physiological excipients. We then sought to examine the effect of IK14004 on Ki67 and CD25 expression in T cells within stimulated PBMC cultures after 72 h. In contrast to isolated T cells cultured in the absence of APCs, in the presence of APCs IK14004 had no effect on the proliferative capacity of either CD4+ T cells (Fig. 1h) or CD8+ T cells (Fig. 1j) whereas the expression of CD25 was reduced in CD4+ T cells (Fig. 1i) and CD8+ T cells (Fig. 1k). Furthermore, no IL-2 could be detected in supernatants either in the absence or presence of IK14004 after 72 h.

### IK14004 supresses DC licensing and maturation

**Figure 2:**
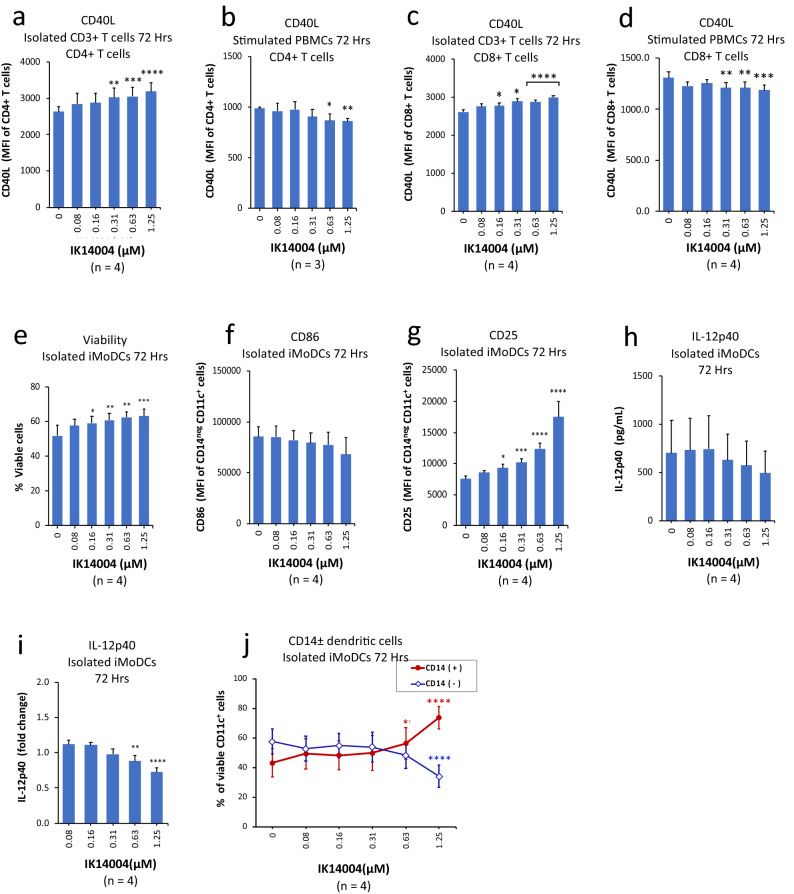
IK14004 suppresses DC licensing and maturation. Immature monocyte-derived dendritic cells (iMoDCs) were prepared as described in the “Methods”. Each tissue culture experiment involving use of either monocytes, PBMCs or isolated CD3+ T cells was performed using triplicate wells (technical replicates) and flow cytometry/ELISA experiments were repeated at least three times (n = experimental replicates) as indicated below each panel. All error bars represent standard error of the mean (SEM). In all experiments the culture duration was 72 h. The compound shown is IK14004 as indicated under each panel. Flow cytometry data are shown as mean fluorescence intensity (MFI). Dot plots and gating strategies are shown in Supplementary Figs. S10 to S17. (**a**) Expression of CD40L in CD4+ T cells within T cell cultures. (**b**) Expression of CD40L in CD4+ T cells within PBMC cultures. (**c**) Expression of CD40L in CD8+ T cells within T cell cultures. (**d**) Expression of CD40L in CD8+ T cells within PBMC cultures. (**e**) Viability of immature monocyte-derived dendritic cell (iMoDC) cultures. (**f**) Expression of CD86 in iMoDC cultures. (**g**) Expression of CD25 in iMoDC cultures. (**h**) IL-12p40 levels in supernatant from iMoDC cultures. (**i**) IL-12p40 production by iMoDCs expressed as fold-change between the highest and lowest IK14004 concentration, respectively, with vehicle-treated cells normalised to 1. (**j**) Reversion of CD14 negative iMoDCs to CD14+ monocytes. Data were analysed using repeated measures (RM) two-way ANOVA with Dunnett’s post-test comparing peptide with vehicle control. *P < 0.05, **P < 0.01, ***P < 0.001, ****P < 0.0001.

The CD40L-CD40 interaction between CD4+ T helper cells and dendritic cells (DCs) is important for DC licensing and maturation, upregulation of DC co-stimulatory molecules (CD80 and CD86), and secretion of cytokines necessary for activation of cytotoxic lymphocytes^32,56^. Given differences observed in the effect of IK14004 on T cells when in the absence and presence of APCs (Fig. 1), this raised the possibility that IK14004-mediated effects on T cells in the presence of APCs might relate to alterations in DC maturation. We therefore compared the effect of IK14004 on CD40L expression in T cells within isolated T cell cultures and T cells within PBMC cultures after 72 h. PBMC and T cell cultures were stimulated with anti-CD3 and anti-CD3/anti-CD28 antibodies, respectively. IK14004 induced a dose-dependent increase in CD40L expression in CD4+ T cells (Fig. 2a) in contrast to decreased expression in the presence of APCs (Fig. 2b). Similarly, CD40L expression was increased in CD8+ T cells within T cell cultures (Fig. 2c) and inhibited in CD8+ T cells within PBMC cultures (Fig. 2d). We then tested the effect of IK14004 on viability of DCs in isolated monocyte-derived DC cultures and this increased significantly in a dose-dependent manner (Fig. 2e). The co-stimulatory molecule, CD86, is constitutively expressed in human peripheral blood DCs in contrast to CD80^57^ and mature DCs express higher levels of CD86 than immature DCs^24^. Given that CD40L expressed on CD4+ T cells contributes to DC maturation^32^ and IK14004 inhibited CD40L expression in PBMC cultures, we next examined the effect of IK14004 on CD86 expression in isolated DC cultures after 72 h and no statistically significant trend was observed (Fig. 2f). The biological function of CD25 during DC maturation remains undefined and considered to act as a potential “sink” for available IL-2^58^. IK14004 induced an increase in CD25 expression in isolated DC cultures after 72 h that approximated 130% (Fig. 2g) compared with an increase in CD4+ T cells within isolated T cell cultures at the same time point that approximated 25% (Fig. 1b). DC maturation also varies with their IL-12-producing capacity^32^ and IK14004 inhibited IL-12p40 production from isolated DC cultures shown as pg/mL (Fig. 2h) and fold-decrease (Fig. 2i). In addition, IK14004 induced reversion of the CD14-negative DC phenotype to CD14+ monocytes after 72 h (Fig. 2j).

### IK14004 enhances expression of activating co-receptors in CD8+ T cells and induces STAT5 activation

**Figure 3:**
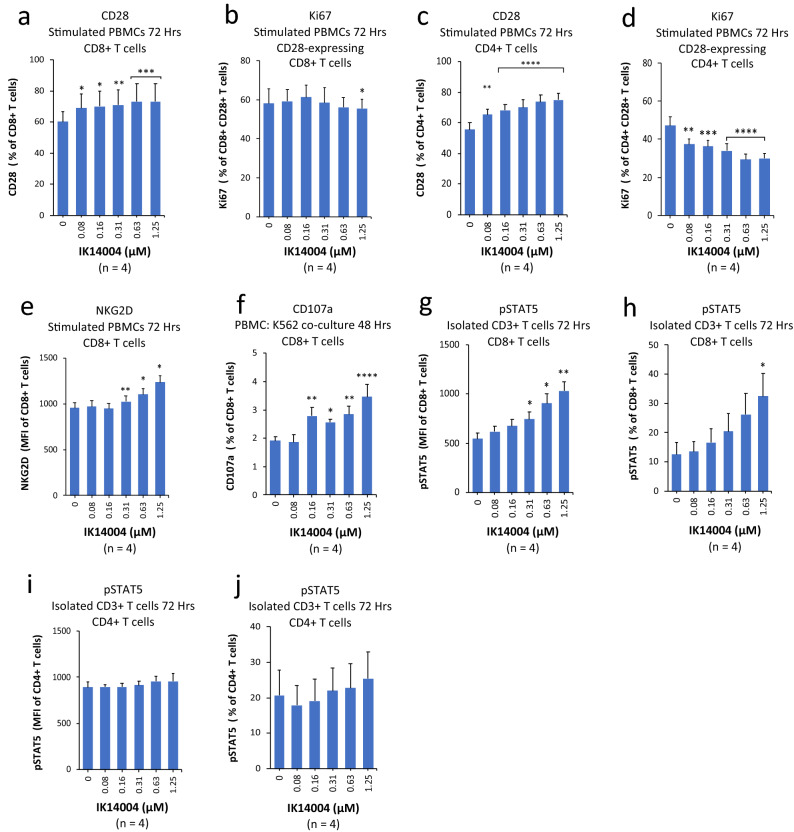
IK14004 enhances expression of activating co-receptors in CD8+ T cells and induces STAT5 activation. Experiments with PBMCs or isolated CD3+ T cells were performed using triplicate wells (technical replicates) for each experiment and flow cytometry experiments were repeated four times (n = experimental replicates) as indicated below each panel. All error bars represent standard error of the mean (SEM). Cells were cultured for 48 h in a granzyme B assay (CD107a) and in all other experiments for 72 h. The granzyme B assay is described in the “Methods”. The compound shown is IK14004 as indicated under each panel. Flow cytometry data are shown as percentages and mean fluorescence intensity (MFI). Dot plots and gating strategies are shown in Supplementary Figs. S18 to S27. (**a**) Percentage of CD28 + CD8 + T cells within PBMC cultures. (**b**) Percentage of Ki67 + CD28 + CD8 + T cells within PBMC cultures. (**c**) Percentage of CD28 + CD4 + T cells within PBMC cultures. (**d**) Percentage of Ki67 + CD28 + CD4 + T cells within PBMC cultures. (**e**) Expression of NKG2D in CD8 + T cells within PBMC cultures. (**f**) Percentage of CD107a + CD8 + T cells within a PBMC : K562 co-culture assay. (**g**) Expression of pSTAT5 in CD8+ T cells within T cell cultures. (**h**) Percentage of pSTAT5-expressing CD8+ T cells within T cell cultures. (**i**) Expression of pSTAT5 in CD4+ T cells within T cell cultures. (**j**) Percentage of pSTAT5-expressing CD4+ T cells within T cell cultures. Data were analysed using repeated measures (RM) two-way ANOVA with Dunnett’s post-test comparing peptide with vehicle control. *P < 0.05, **P < 0.01, ***P < 0.001, ****P < 0.0001.

The activating co-receptors on T cells include CD28 and the natural-killer group 2, member D (NKG2D) receptor, which are expressed in approximately half and all mature CD8+ T cells, respectively^59^. In initial studies we sought to determine the effect of IK14004 on CD28 expression in CD4+/CD8+ T cells within PBMC cultures after 72 h including the effect of IK14004 on the proliferative capacity of CD28-expressing cells. PBMC and T cell cultures were stimulated with anti-CD3 and anti-CD3/anti-CD28 antibodies, respectively. IK14004 enhanced the expression of CD28 in CD8+ T cells (Fig. 3a) with a small inhibitory effect on Ki67 expression seen only at the highest concentrations (1.25 µM) (Fig. 3b). IK14004 also enhanced the expression of CD28 in CD4+ T cells (Fig. 3c) but induced a marked dose-dependent decrease in proliferative capacity of CD28-expressing CD4+ T cells commencing at the lowest concentration (0.08 µM)(Fig. 3d). We next assessed the effect of IK14004 on expression of NKG2D after 72 h and this increased in a dose-dependent manner (Fig. 3e). The granzyme B (GrB) degranulation marker, CD107a, is associated with target cell killing by cytotoxic lymphocytes and considered an immune signature for CD8+ T cell activation^60^. Given the stimulatory effect of IK14004 on activating co-receptors in CD8+ T cells we then assessed the effect of IK14004 on GrB expression in CD8+ T cells in a PBMC/K562 co-culture assay and observed a significant increase in the proportion of CD107a-expressing CD8+ T cells (Fig. 3f). To further assess CD8+ T cell effector function we sought to compare STAT5 activation in CD4+/CD8+ T cells within CD3+ T cell cultures after 72 h because this transcription factor is critical in the maintenance of effector CD8+ T cells responses^61^. IK14004 markedly activated STAT5 (Fig. 3g) and increased the proportion of CD8+ T cells that expressed activated STAT5 (Fig. 3h) in contrast to no effect on the level of STAT5 activation in CD4+ T cells (Fig. 3i) and no effect on the proportion of CD4+ T cells that expressed activated STAT5 (Fig. 3j).

### IK14004 regulates the IL-12p70 : IL-12p40 ratio both in the absence and presence of APCs

**Figure 4:**
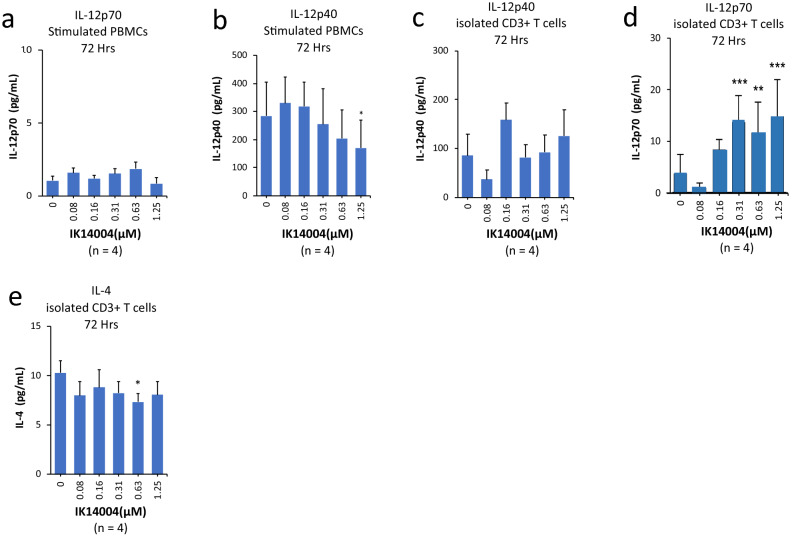
IK14004 regulates the IL-12p70: IL-12p40 ratio both in the absence and presence of APCs. Experiments with PBMCs or isolated CD3+ T cells were performed using triplicate wells (technical replicates) for each experiment and ELISA experiments were repeated four times (n = 4) as indicated below each panel. All error bars represent standard error of the mean (SEM). In all experiments the culture duration was 72 h. The compound shown is IK14004 as indicated under each panel. (**a**) IL-12p70 levels in supernatant from PBMC cultures. (**b**) IL-12p40 levels in supernatant from PBMC cultures. (**c**) IL-12p40 levels in supernatant from T cell cultures. (**d**) IL-12p70 levels in supernatant from T cell cultures. (**e**) IL-4 levels in supernatant from T cell cultures. Data were analysed using repeated measures (RM) two-way ANOVA with Dunnett’s post-test comparing peptide with vehicle control. *P < 0.05, **P < 0.01, ***P < 0.001, ****P < 0.0001.

DC maturation is associated with IL-12p40 production^32^ and taken together with IK14004-mediated suppression of CD86 and destabilisation of DCs, (Fig. 2f) and (Fig. 2j) respectively, we sought to compare the effects of IK14004 on levels of IL-12p70 and IL-12p40 in supernatants from anti-CD3 stimulated PBMC cultures after 72 h. Production of IL-12p70 was low but unaffected by IK14004 (Fig. 4a) in contrast to suppression of IL-12p40 production (Fig. 4b). Peripheral blood CD4+ T cells have been reported to express intracellular IL-12^62^ and we therefore compared the effect of IK14004 on production of both IL-12 forms from anti-CD3/anti-CD28 stimulated T cell cultures at the same time point. Much higher levels of IL-12p40 than IL-12p70 are normally produced^63^ and IK14004 did not affect IL-12p40 production (Fig. 4c) in contrast to IL-12p70 which increased in a dose-dependent manner (Fig. 4d). The IL-12p40 ELISA assay does not differentiate between the two forms of IL-12, i.e., IL-12p70 comprising p40 and p35 disulphide-linked chains and the IL-12p40 monomers/dimers. Notably, IL-23 also consists of a p40 subunit combined with a p19 chain; however, IL-23 could not be reliably assessed in culture supernatants because levels were mostly below detection limits. Although it is theoretically possible that increased production of IL-12p70 by isolated CD3+ T cells could have been due to contaminating myeloid cell subsets we considered this unlikely given IK14004-mediated inhibition of IL-12p40 production by DCs (Fig. 2h and Fig. 2i). The cytokine interleukin-4 (IL-4) is a Th2 cell product and in the presence DCs this acts as a co-factor for T helper cell-mediated IL-12p70 production^64^. Given our finding that IK14004 enhanced IL-12p70 production in the absence of APCs, this raised the possibility that IL-4 produced by T cells may also play a role in IK14004-induced IL-12p70 production in the absence of APCs. Exposure of isolated CD3+ T cell cultures to IK14004 for 72 h appeared to induce a slight inhibitory trend in IL-4 production which was statistically significant only at one of the higher concentrations (Fig. 4e).

### IK14004 inhibits IFN-γ expression and increases the proportion of T regulatory (Treg) cells

**Figure 5:**
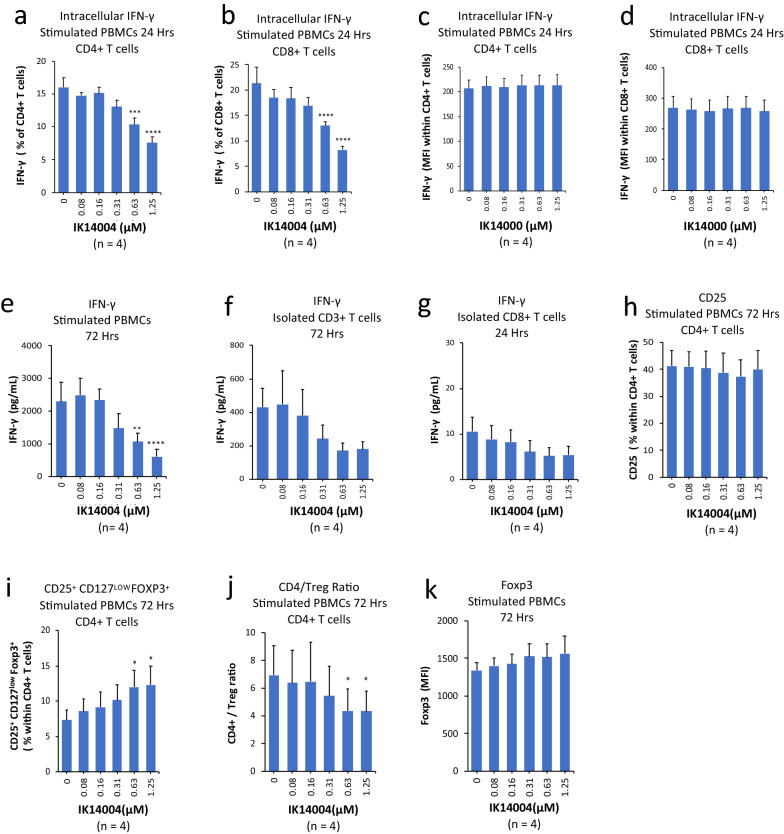
IK14004 inhibits IFN-γ expression and increases the proportion of T regulatory (Treg) cells. Stimulated PBMCs, isolated CD3+ T cells, or isolated CD8+ T cells were cultured in triplicate wells in each experiment. Each experiment was repeated four times (n = 4) as shown below each panel. Both flow cytometry and ELISA assays were conducted to assess IFN-γ expression either after 24 or 72 h. The compounds tested, IK14004 and IK14000, are listed under each panel. All error bars represent standard error of the mean (SEM). Flow cytometry data are shown as mean fluorescence intensity (MFI) and percentage values as indicated in the panels. Dot plots and gating strategies are shown in Supplementary Figs. S28 to S35. (**a**) Percentage of CD4+ T cells expressing intracellular IFN-γ within PBMC cultures exposed to the lipidic peptide, IK14004, for 24 h. (**b**) Percentage of CD8+ T cells expressing intracellular IFN-γ within PBMC cultures exposed to IK14004 for 24 h. (**c**) Intracellular expression of IFN-γ in CD4+ cells within PBMCs cultured for 24 h in the presence of RSKAKNPLYR (IK14000) that lacks the dodecanoic acid moiety. (**d**) Intracellular expression of IFN-γ in CD8+ cells within PBMC cultures exposed to IK14000 for 24 h. (**e**) Levels of IFN-γ within supernatants from PBMC cultures exposed to IK14004 for 72 h. (**f**) Levels of IFN-γ within supernatants from isolated CD3+ T cell cultures exposed to IK14004 for 72 h. (**g**) Levels of IFN-γ within supernatants from isolated CD8+ T cell cultures exposed to IK14004 for 24 h. (**h**) Percentage of CD25 + CD4 + T cells within PBMC cultures exposed to IK14004 for 72 h. (**i**) Percentage of CD4 + CD25 + Foxp3 + Treg cells within PBMC cultures exposed to IK14004 for 72 h. (**j**) The total CD4+: Treg cell ratio within PBMC cultures exposed to IK14004 for 72 h. (**k**) Expression levels of Foxp3 in CD25 + CD4 + T cells within stimulated PBMC cultures exposed to IK14004 for 72 h. Data were analysed using repeated measures (RM) two-way ANOVA with Dunnett’s post-test comparing peptide with vehicle control. *P < 0.05, **P < 0.01, ***P < 0.001, ****P < 0.0001.

An IFN-γ/IL-12 amplifying autoregulatory loop has been proposed based on IFN-γ-mediated induction of IL-12p40 mRNA^8^. Taken together with our observation that IK14004 suppresses IL-12p40 production in DCs (Fig. 2h,i) and PBMCs (Fig. 4b), we sought to determine the effect of IK14004 on intracellular IFN-γ expression in CD4+/CD8+ T cells within anti-CD3 stimulated PBMC cultures after 24 h. The proportion of CD4+ T cells expressing IFN-γ was significantly reduced in the presence of IK14004 (Fig. 5a) as was the proportion of CD8+ T cells (Fig. 5b). Given that the dodecanoic acid moiety within IK14004 was essential for IL-2 production (Fig. 1g), we therefore examined the effect of the unconjugated peptide sequence RSKAKNPLYR (IK14000) on IFN-γ expression. Intracellular expression of IFN-γ in the presence of IK14000 remained unchanged in CD4+ T cells (Fig. 5c) and in CD8+ T cells (Fig. 5d). To establish that the effect of IK14004 on expression of IFN-γ was mirrored by cytokine production in cell culture supernatants, we next tested the effect of IK14004 on IFN-γ produced by PBMC and anti-CD3/anti-CD28 stimulated T cell cultures by means of ELISA assays after 72 h. IK14004 inhibited IFN-γ production from PBMCs (Fig. 5e), isolated CD3+ T cell cultures (Fig. 5f) and cultures of isolated CD8+ T cells (Fig. 5g). IFN-γ is known to drive Treg fragility^65^ and given our findings, we examined the effect of IK14004 on Tregs within stimulated PBMC cultures after 72 h. Exposure of anti-CD3-stimulated PBMCs to IK14004 did not alter the total proportion of CD25-expressing CD4+ T cells (Fig. 5h). The proportion of CD25-expressing cells that also expressed Foxp3 increased in the presence of IK14004 (Fig. 5i) which was reflected in the CD4/Treg ratio at higher IK14004 concentrations (Fig. 5j). However, this was not associated with a statistically significant increase in the level of Foxp3 expression (Fig. 5k).

### Molecular mechanisms of action of IK14004

**Figure 6:**
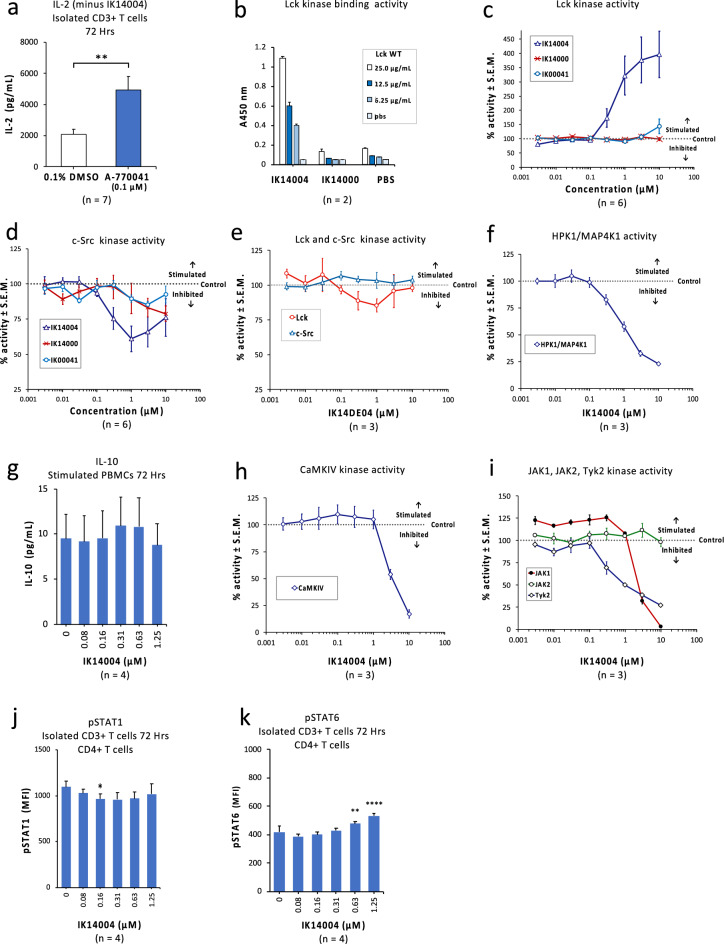
Molecular mechanisms of action of IK14004. In cell-based studies the culture duration was 72 h and non-cell-based Lck binding and kinase profiling assays were performed as described in the “Methods”. IL-2 and IL-10 levels in supernatants obtained from anti-CD3/anti-CD28 stimulated CD3+ T cells and anti-CD3 stimulated PBMC cultures, respectively, were determined by means of ELISA assays (triplicate technical replicates and 4 experimental replicates). Activation of STAT1 and STAT6 in CD4+ T cells within CD3+ T cell cultures exposed to IK14004 was assessed by flow cytometry (4 experimental replicates). Flow cytometry data are shown as mean fluorescence intensity (MFI) and all error bars represent standard error of the mean (SEM). Dot plots and gating strategies are shown in Supplementary Figs. S36 to S37. (**a**) IL-2 levels in supernatant obtained from isolated CD3+ T cell cultures exposed to either 0.1% DMSO (control) or the Lck inhibitor, A-770041 (0.1 µM). (**b**) A representative plot from duplicate non-cell-based ELISA experiments (each with 3 technical replicates) performed to assess binding of IK14004 to Lck wild-type (Lck-WT) cells at various dilutions. (**c**) Lck activity in the presence of IK14004, IK14000 and IK00041. (**d**) c-Src activity in the presence of IK14004, IK14000 and IK00041. (**e**) Lck and c-Src activity in the presence of IK14DE04 containing acidic amino acid residues. (**f**) HPK1 activity in the presence of IK14004. (**g**) Levels of IL-10 within supernatants from PBMC cultures exposed to IK14004 for 72 h. (**h**) CaMKIV activity in the presence of IK14004. (**i**) Activities of JAK1, JAK2 and Tyk2 in the presence of IK14004. (**j**) Expression of pSTAT1 in CD4+ T cells within T cell cultures exposed to IK14004. (**k**) Expression of pSTAT6 in CD4+ T cells within T cell cultures exposed to IK14004. Data derived from testing the Lck inhibitor, A-770041 (7 experimental replicates each comprising 3 technical replicates) were analysed using a paired t-test **P < 0.01. Data for all other cell-based assays were analysed using repeated measures (RM) two-way ANOVA with Dunnett’s post-test comparing peptide with vehicle control. *P < 0.05, **P < 0.01, ***P < 0.001, ****P < 0.0001.

Given the role of Lck in upstream signalling upon TCR activation^34,35^ which leads to IL-2 production^10,11^, we tested the effect of a known Lck inhibitor, A-770041 on IL-2 expression within supernatants from stimulated T cell cultures after 72 h. A concentration of 0.1 uM was chosen given reported IC_50_ values of 147 nM and 80 nM for inhibition of Lck activity and IL-2 production in vitro, respectively^66,67^. Surprisingly, A-770041 induced IL-2 production (Fig. 6a) at a comparable nanomolar concentration to IK14004 (0.16 uM) but to a much greater level than in the presence of IK14004 (Fig. 1f). This raised the possibility that IK14004 binds to Lck. Using Lck-wild-type protein with a C-terminal mCherry reporter (see Methods), binding of IK14004 to Lck was confirmed (Fig. 6b). In the absence of the dodecanoic acid moiety, binding of peptide IK14000 was no different to the negative control, i.e., phosphate-buffered saline (PBS) (Fig. 6b). Induction of IL-2 by the Lck inhibitor, A-770041, suggested that IK14004 could be a Lck inhibitor. We therefore tested the effect of IK14004 and its two components, i.e., IK14000 and IK00041, on Lck activity in a non-cell-based assay. Surprisingly, Lck activity was markedly enhanced whereas neither IK14000 nor IK00041 had an effect (Fig. 6c). Given that SFK members share a common domain structure^68^ and that RSKAKNPLYR has previously been reported to inhibit c-Src activity^53^ we next sought to compare the effects of IK14004, RSKAKNPLYR (IK14000) and the dodecanoic acid moiety (IK00041) on the activity of c-Src using a non-cell-based kinase profiling approach. A combination of peptide plus dodecanoic acid, i.e., IK14004, was the most effective inhibitor of c-Src activity at concentrations below 3 µM (Fig. 6d). Since the peptide RSKAKNPLYR (IK14000) in combination with the lipid moiety was necessary for activating Lck and inhibiting c-Src (Fig. 6c,d), we sought to determine whether basic residues (arginine and lysine) within RSKAKNPLYR play a role in these events. Substitution for acidic residues, i.e., aspartate and glutamate (sequence DSEAENPLYD combined with the lipid moiety and designated IK14DE04), abolished the effects of IK14004 on not only Lck but also c-Src activity (Fig. 6e).

The germinal centre kinase, HPK1 (MAPKIV) is a negative-regulator of the TCR signalling pathway^69^ and activation of HPK1, which is required for limiting T cell responsiveness, is dependent on the presence of Lck-mediated signalling pathways^36^. We therefore chose to examine the effect of IK14004 on HPK1 activity in a non-cell-based assay and HPK1 activity was inhibited with an IC_50_ approximating 1 µM (Fig. 6f). IL-10 is produced mainly by Tregs and dampens the pro-inflammatory responses of IFN-γ^70^. Given IK14004-mediated suppression of IFN-γ (Fig. 5), we then tested the effect of IK14004 on IL-10 production after 72 h in an anti-CD3 stimulated PBMC assay. IK14004 had no effect on IL-10 production (Fig. 6g) which raised the possibility of alternative IL-10-independent signalling pathways involving calcineurin-regulated NFAT translocation that are known to regulate transcription of IFN-γ^46,47^. Using a non-cell-based assay, IK14004 was found to inhibit CaMKIV activity with an IC_50_ of 3 µM (Fig. 6h). Amongst the Janus kinase (JAK) family members, Tyk2, also plays a critical role in regulating IFN-γ expression^49^ and ligand engagement of the IFN-y receptor leads to activation of both JAK1 and JAK2 which serve as a docking site for STAT1^71^. We thus used non-cell-based assays to test the effect of IK14004 on activity of JAKs, and the activities of Tyk2 and JAK1 were inhibited with IC_50_ values of 1 µM and 3 µM, respectively, whereas the activity of JAK2 remained unaltered (Fig. 6i). Given that STAT1 suppresses STAT6^71^ and STAT6 is phosphorylated by IL-2 signalling in Th1 cells^72^ we next examined the effect of IK14004 on activity of STAT1 and STAT6 in anti-CD3/anti-CD28 stimulated T cell cultures after 72 h. No dose-dependent trend was observed for STAT1 (Fig. 6j) whereas activation of STAT6, i.e., phosphorylation, increased in a dose-dependent manner (Fig. 6k).

## Discussion

**Figure 7:**
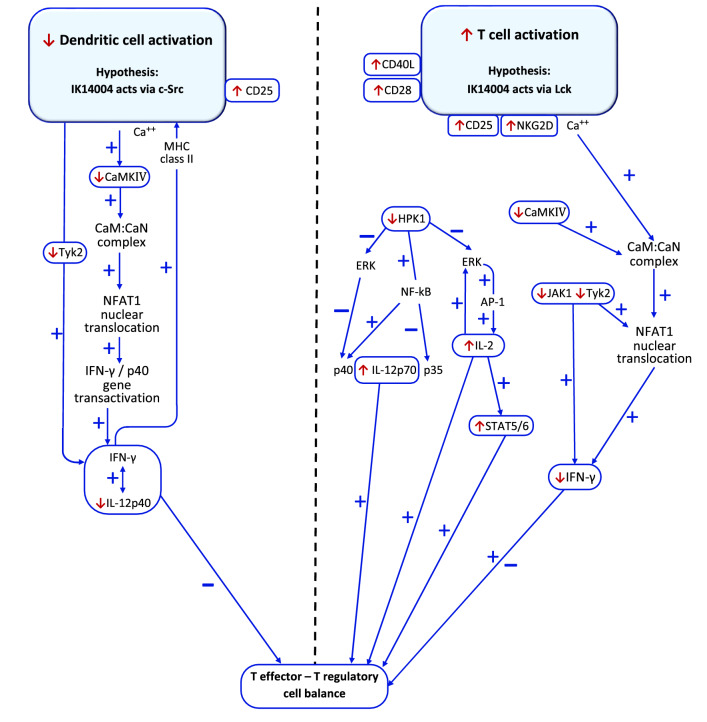
Schematic illustrating immunomodulatory effect of IK14004 and putative effects on kinase activities relevant to maintaining a balanced immune response. Known regulatory pathways are shown in blue arrows with blue ± symbols representing stimulatory/inhibitory effects, respectively. IK14004 effects are shown as upward or downward red arrows representing stimulatory/inhibitory effects, respectively. Whether the contrasting effects of IK14004 on activity of Lck and c-Src in non-cell-based assays are physiologically relevant remains to be established in cell-based systems. IK14004 suppresses DC activation and production of IL-12p40 which regulates IFN-γ expression in a positive feedback loop. T cell production of IL-2 and IFN-γ is uncoupled and IK14004 alters the IL-12p70 : IL-12p40 ratio which has implications for maintenance of functional CD4+ Treg cell populations. IK14004 increases the proportion of Treg cells and induces expression of activating co-receptors in CD8+ T cells coupled with enhanced granzyme B expression. Taken together with STAT5 activation in CD8+ but not CD4+ T cells and changes in cytokine expression, these divergent effects suggest a role for IK14004 in maintenance of balanced immune responses involving T cell-DC interactions.

An integrated understanding of how IL-2 and TCR co-stimulatory signals combine to control homeostasis in distinct tissue locations is lacking^11^. The effects of IK14004 on cytokine production are shown in the Schematic (Fig. 7) together with putative effects on kinase-mediated signalling pathways involved in production of these cytokines. Maturation of DCs involves CD40L-CD40 interactions dependent upon T helper expression of CD40L^21,22^. CD40L expression is partly dependent at later time points, i.e., 48 h, on IL-2-mediated signalling which has been shown to be inhibitable in vitro by daclizumab, a humanised anti-CD25 mAb^73^. It is therefore possible that IK14004-mediated inhibition of CD25 and CD40L expression at such a later time point in PBMC cultures is not only related, but also secondary, to a limited supply of IL-2. IL-2 is known to induce expression of CD25^16^ and in the absence of APCs, IK14004 induces expression of CD40L and IL-2 which is associated with induction of CD25 expression in CD4+ T cells and enhanced proliferative capacity. Notwithstanding release of IL-2 by DCs that can bind in an autocrine manner to CD25 expressed on the surface of DCs^74^, we suggest that the large increase in CD25 expression in DCs in the presence of IK14004 (Fig. 2g) serves as an IL-2 “sink” as has been reported^58^. The development of such a “sink” induced over 72 h by IK14004 could account for the failure to detect IL-2 in PBMC supernatants at that time-point associated with suppression of both CD40L and CD25 in CD4+ T cells and lack of effect on proliferative capacity.

IK14004 enhances expression of IL-2, CD25 and CD40L in purified T cell cultures and we cannot exclude the possibility that IK14004-mediated IL-12p70 and IL-2 production plays a role in increased CD40L expression in T cells that are not in contact with APCs because recombinant IL-12 and IL-2 have been shown to synergistically induce optimal CD40L expression in T cells^27^. IFN-γ production by T cells has also been attributed, in part at least, to IL-2-mediated signalling given inhibition of IFN-γ production by T cells in the presence of anti-CD25 mAb^73^. We do not believe that IK14004-mediated suppression of IFN-γ production acts via this pathway given the uncoupling of IL-2 and IFN-γ production by IK14004 in isolated T cell cultures. Furthermore, if anti-CD25 abrogates IL-12p70 production by PBMCs as suggested by these investigators^73^, one might expect suppression of IL-12p70 production from PBMCs in the presence of IK14004 rather than no effect. Instead, a possible reason for IK14004-mediated inhibition of IFN-γ could be alteration in the IL-12p70 : IL-12p40 ratio either in the presence or absence of APCs when exposed to IK14004. For example, IL-12p40 and IFN-γ positively regulate each other and an IFN-γ/IL-12 amplifying loop has been suggested based on IFN-γ-mediated induction of IL-12p40 mRNA^8^. To the best of our knowledge, we provide the first evidence of selective induction of IL-12p70 protein expression, but not IL-12p40, from isolated T cells when exposed to an external agent. We suggest that in the presence of APCs, this IL-12 ratio is altered by the inhibitory effect of IK14004 on IL-12p40 production by DCs which may be secondary to IK14004-mediated DC destabilisation resulting in an even more immature, tolerogenic DC phenotype. In the absence of APCs, the preference of IK14004 for IL-12p70 induction rather than IL-12p40 may be due to indirect effects of IK14004 on both ERK and NF-kB signalling pathways given negative feedback by NF-kB on the p35 subunit of IL-12p70 via a calcium-dependent mechanism in contrast to ERK which mediates negative feedback on regulation of p40, but not p35^42^. Another possible reason for IK14004-mediated changes to the IL-12p70 to p40 ratio may be that IK14004 indirectly exerts independent control of the expression of genes for IL-12p35 and IL-12p40. IFN-γ production in effector Th1 cells is also inhibited by IL-4^75^. While our observation of decreased IFN-γ production associated with IK14004-mediated suppression of IL-12p40 raised the possibility of IL-4 involvement, this seems unlikely given slight inhibition of IL-4 production by T cells rather than stimulation in the presence of IK14004 although downstream signalling from IL-4 has not been excluded.

IL-12p40 inhibits IL-12p70 activity^76^ and IL-12p40 exerts an inhibitory effect on Tregs^29^. Taken together with the contribution by IL-12p70 towards induction of Th1-like Tregs^28^, an alteration in the IL-12p70 : IL-12p40 ratio would appear consistent with promotion of Tregs by IK14004. However, this begs the question of how such a Treg-promoting effect can be reconciled with IK14004-induced inhibition of CD25 expression in CD4+ T cells in the presence of APCs? It has been suggested that the paradoxical benefit in autoimmune flare-ups from anti-CD25 therapies may be due to suppression of CD4 + CD25 + Foxp3 negative cells associated with more effective cleavage of cell-surface CD25 from non-Foxp3-expressing CD4+ T cells compared with Tregs following T cell activation^77^ and we cannot exclude the possibility of more effective cleavage of CD25 from non-Foxp3-expressing cells induced by IK14004. Secondly, maintenance of Tregs also involves CD28 besides IL-2/CD25-mediated signalling^11^. If the marked inhibition in proliferative capacity of CD28-expressing CD4+ T cells induced by IK14004 (Fig. 3d) is dominant within the CD4+ T effector cell population, then this could lead to a relative increase in the proportion of CD28-expressing CD4 + Tregs. In addition, signals mediated by STAT6 lead to efficient Foxp3 expression^78^ which may explain why autoimmune pathologies are exacerbated in the absence of STAT6^79^. Given that STAT6 is phosphorylated by IL-2 signalling in Th1 cells^72^ we suggest that IK14004-mediated STAT6 activation could be involved in maintaining Foxp3 expression depending on the availability of IL-2. Furthermore, if IK14004 induces a more immature DC phenotype consequent upon DC destabilisation associated with decreased IL-12p40 production, then this could also play a role in sustaining Tregs. Mature DCs express higher levels of CD86 than immature DCs^24^ and stimulation of Tregs via CD86 inhibits their suppressive function which improves in the presence of immature DCs^24^. Notably, CD86-silenced DCs have been associated with immune tolerance and reduced IFN-γ levels in a mixed lymphocyte reaction model^25^. The downward trend in CD86 expression in the presence of IK14004 was not significant and a reflection of donor-to-donor variation.

CD4+ T cells are major producers of IL-2 which acts in a paracrine manner to support effector and memory CD8+ T cell differentiation^17^. In addition, robust CD8+ effector T cell responses are known to be generated by licensing DCs through CD40L expressed on CD4+/CD8+ T cells and then priming CD8+ T cells^26^. CD4+ T cells can also help CD8+ T cells in the absence of CD40L-mediated DC licensing and CD40L signals required by CD8+ T cells do not appear to originate from an autologous source^80^. It is therefore possible that enhanced CD40L expression on CD4+ T cells induced by IK14004 in the absence of APCs could lead to CD4+ T cell-mediated priming of adjacent CD8+ T cells. Furthermore, the TCR co-receptor, CD28, is expressed on T effector cells largely upon TCR activation^3^ and functionality of the natural killer c-type lectin receptor, NKG2D, expressed on activated CD8+ T cells^81^ requires activation of the TCR^59^. IK14004 enhances expression of both activating co-receptors in CD8+ T cells including granzyme B expression and, given the relevance of these co-receptors in cancer immunotherapy^82,83^, we cannot exclude the possibility of an indirect immune-related effect of IK14004 on tumour growth. Furthermore, activation of STAT5 is required for effector CD8+ T cell responses^61^ and STAT5 has dramatically divergent effects on CD8+ T cells versus CD4+ T cells leading to expansion of CD8+ memory-like T cells and CD4 + CD25+ regulatory T cells^84^. Lack of effect of IK14004 on STAT5 activation in CD4+ T cells in contrast to CD8+ T cells suggests that IK14004-mediated support of Tregs may be independent of STAT5 signalling pathways.

There are several limitations to our study that relate to absence of cell-based data with respect to interactions between IK14004 and intracellular molecules and the IK14004-mediated effects on the activity of kinases. Firstly, we have not determined whether IK14004 binds to other targets which could be established by means of IK14004 pull-down assays in stimulated T cells combined with mass spectrometry. Secondly, we have not confirmed that the effects of IK14004 on the activity of Lck and other kinases are physiologically relevant. In the case of Lck, the role of Lck in upstream signalling upon TCR activation^34,35^ associated with IL-2 production^10^ is well established. We have observed that induction of IL-2 and CD25 expression requires the presence of the dodecanoic acid moiety within IK14004. Lck activation by IK14004 and binding to Lck also requires the presence of this moiety. Taken together, this raises the possibility that IK14004-mediated Lck activation could be physiologically relevant notwithstanding that this has not been established in a cell-based system.

On the other hand, IK14004 appears to behave like a Lck inhibitor with respect to IL-2 induction when compared with the known Lck inhibitor, A-770041^66^. Notably, our study and that of Stachlewitz and colleagues^66^, who reported in vitro inhibition of IL-2 production in the presence of A-770041 using heparinised whole blood exposed to both anti-CD3 plus phorbol myristate acetate (PMA), were conducted under very different experimental conditions. This highlights the importance of evaluating the effects of Lck inhibition on cytokine production in a range of relevant cell-based systems given the possibility of Lck-independent T cell activation and the existence of two pools of Lck with opposing effects on TCR-mediated signalling^85^. In addition to this level of complexity, the Tyr192 phosphorylation site in Lck fine-tunes Lck activation which may serve to maintain a pool of already active Lck in resting T cells^86^. The importance of comparing IK14004 effects on all three tyrosine residues in Lck, i.e., Tyr394, Tyr505, and Tyr192, is emphasised given the paradoxical phosphorylation of both activating and inhibitory Lck tyrosine residues upon stimulation of T cells^87^. However, it is the magnitude of phosphorylation at the activation site, Tyr394, that is thought to override the inhibitory residue^87^ while Tyr192 controls the activity of both residues^86^. Hence, Lck is critically dependent on Tyr394 phosphorylation in its active site loop for its kinase activity^88^ and assymetrical dimerization of Lck with the Tyr394 activation loop of one monomer in the other monomer’s active site is associated with *trans*-autophosphorylation of Lck which is believed to be crucial for Lck activation^89^. We suggest that IK14004 induces Lck dimerization via electrostatic interactions involving its peptide component including binding of the lipid moiety to a hydrophobic region in Lck. Differences exist with respect to the unique domain (UD) and lipid-binding regions of Lck and c-Src^68^ and we do not know how IK14004 contributes to inhibition of c-Src activity. Combination of RSKAKNPLYR with the lipid moiety could interfere with c-Src dimerization which is known to enhance autophosphorylation^90^ and the binding domains in Lck and c-Src may be sufficiently different to account for the observed specificity of IK14004.

A further limitation to our study is that we have not examined the effects of IK14004 on Lck’s regulatory phosphorylation sites (Tyr394, Tyr505 and Tyr192) including phosphorylation of molecular targets of Lck, e.g., ZAP70, CD3ζ, Lat, PLCy1 and ERK1/2 as well as calcium flux under both steady state conditions and upon TCR stimulation. Discrimination between IK14004 effects on upstream versus downstream signalling upon TCR stimulation and Lck activation could be established using Lck-expressing and Lck-deficient cells stimulated either through the TCR or by means of PMA plus Ionomycin. However, this may not exclude the possibility of Lck becoming activated by bottom-up signalling. Such studies could also help to resolve conflicting reports of large increases in IL-2 production with Lck knock-down^91^ versus suppression of IL-2 production in the presence of SFK inhibitors^92^. Furthermore, it would be interesting to compare effects of IK14004 on phosphorylation of proximal signalling molecules involved in TCR signalling and cytokine production with data recently reported for a 10 mer peptide linked to a non-lipid cell-entry moiety^93^. These investigators incubated effector T cells with a peptide concentration much higher than that used in our study resulting in inhibition of both IL-2 and IFN-γ^93^. Such comparisons could shed light on how Lck regulates the discrimination between strong and weak agonists at the TCR.

We acknowledge that there are dose-level discrepancies between cell-based immunomodulatory effects detected at the highest concentration of IK14004 (1.25 µM) and the higher IC_50_ values of 3 µM for inhibition of CaMKIV and JAK1 activities in non-cell-based assays. While it remains possible that different immunomodulatory effects could occur at higher concentrations, following bi-weekly administration of IK14004 to mice over two weeks, the in vitro enhancement of CD25 and IL-2 expression in the presence of IK14004 is re-capitulated within splenocyte preparations (M. Agrez, manuscript in preparation). It is also important to establish whether the inhibitory effects of IK14004 on activities of HPK1, JAKs and CaMKIV, are mirrored in cell-based systems. This could help explain the paradoxical dependency of HPK1 catalytic activity on Lck while maintaining the TCR activation threshold^36^ including involvement of HPK1 in the pathogenesis of both autoimmune diseases and cancer^94,95^. Interestingly, IK14004 had no effect on the activity of JAK2 in contrast to Tyk2 which could be relevant to the association between IL-12 receptor subunits and JAKs. For example, Tyk2 is associated with the IL-12Rβ1 chain required for high-affinity binding to the IL-12p40 subunit while the IL-12Rβ2 chain that recognises IL-12p70 is associated with JAK2^96^. Moreover, IL-10 and IFN-γ are negatively correlated to each other^70^. While no inhibitory effect of IK14004 on IL-10 production was identified to account for suppression of IFN-γ, inhibition of CaMKIV could be a contributory factor downstream of IL-10 given the dependency of IFN-γ and IL-12 on calcineurin-regulated NFAT translocation^43,44^. Notably, a direct interaction between SFKs and calmodulin (CaM) has been reported for c-Src^50^. Hence, the convergence of downstream signalling pathways in DCs that lead to NFAT activation^97^ highlights the relevance of establishing whether IK14004 inhibits the activity of both c-Src and CaMKIV in cell-based systems.

In summary, IK14004 represents a new immunomodulatory agent that suppresses DC activation and production of IL-12p40 and IFN-γ. T cell production of IL-2 is uncoupled from IFN-γ and the IL-12p70 : IL-12p40 ratio is altered regardless of the presence or absence of APCs. The lipidic peptide enhances expression of activating co-receptors associated with effector CD8+ T cells while also increasing the proportion of Foxp3-expressing CD4+ T regulatory cells. This compound induces divergent effects on the immune system with potential to promote a balanced immune response upon T cell activation. Taken together, IK14004 offers an opportunity to gain further insights into the complexity of the T cell-DC nexus which may have relevance to autoimmune-related side effects associated with cancer immunotherapy.

## Materials and methods

All methods were carried out in accordance with relevant guidelines and regulations. Buffy coat samples from healthy human donors were obtained from Research Donors Limited via Cambridge BioScience. Ethics approval was granted by the Black Country Research Ethics Committee under REC reference 19/WM/0260 and informed consent was obtained in accordance with the Helsinki Declaration.

### Peptide synthesis

Peptides and lipidic peptides were manufactured by Auspep (Melbourne, Australia) using solid phase peptide synthesis with Fmoc protected amino acid building blocks. Four (2)Adod [(*S*)-2-aminododecanoic acid] residues (Watanabe Chemical Industries LTD, Japan) were coupled sequentially onto a Rink AM resin. Then RSKAKNPLYR-(2)Adod-(2)Adod-(2)Adod-(2)Adod-amide was assembled by sequential addition of each amino acid onto the [(2)Adod]_4_-resin as were the lipidic peptides containing substituted amino acid residues. Once synthesis was completed the lipidic peptides were globally deprotected and cleaved from the resin liberating the crude, C-terminally amidated lipidic peptides. These were purified and salt exchanged to acetate by RP-HPLC (C18) to a purity of > 95%. The product structures were confirmed by mass spectroscopy and amino acid analyses.

### Cell cultures and assays

Preparations of peripheral blood mononuclear cells (PBMCs) and isolated T cells from buffy coat samples were performed using SepMate tubes, EasySep selection and enrichment kits, Lymphoprep, RoboSep Buffer, and EasySep magnets (STEMCELL Company).

PBMCs were resuspended in RPMI-10 (RPMI-1640; ThermoFisher) supplemented with 10% heat inactivated Foetal Bovine Serum (LabTech), 100 U/mL penicillin, 100 µg/mL streptomycin (ThermoFisher), 2 mM l-glutamine (ThermoFisher), and 50 µM β-mercaptoethanol (ThermoFisher) at 1 × 10^6^ cells/mL and plated at a density of 1 × 10^5^ per well (100 µL) in 96-well, flat-bottom culture plates. PBMCs were stimulated with 1 µg/mL of soluble anti-CD3 (BioLegend). The lipidic peptide, IK14004, and IK14000, i.e., RSKAKNPLYR minus conjugation to [{2}Adod]_4_, were solubilised as a 1 mM stock solution in sterile milliQ water (Lonza) and added to wells at a final volume of 50 µL per well together with soluble anti-CD3 (1 µg/mL final, 50 µL per well) (Biolegend). To test the effect of peptides, cells were cultured for 24–72 h at 37 °C and 5% CO_2_. Vehicle controls in peptide-based experiments comprised 0.13% sterile milliQ water in culture medium whereas 0.1% DMSO in culture medium was used as vehicle control for comparison with the Lck inhibitor, A-770041, in the absence of peptides.

CD3^+^ T cells were isolated from PBMCs by negative selection using immune-magnetic separation (Stem cell kits), resuspended in complete medium as used for PBMCs at 0.5 × 10^6^/mL and plated at a density of 5 × 10^4^ per well (100 µL) in 96-well, flat bottom culture plates. CD4^+^ and CD8^+^ T cell populations were isolated by immunomagnetic separation and resuspended in RPMI-10 at 0.5 × 10^6^/mL with a plating density of 0.5 × 10^5^ per well. Either the lipidic peptide, IK14004, or IK14000 (RSKAKNPLYR) was added to wells at a final volume of 50 µL per well, together with anti-CD3 anti-CD28 coated Dynabeads (ThermoFisher) at a 4:1 cell:bead ratio (1.25 × 10^4^/well, 50 µL volume) and cells cultured for 72 h at 37 °C and 5% CO_2_.

Dendritic cells (DCs) were induced from CD14+ monocytes (without CD16 depletion) that had been isolated from PBMCs using immune-magnetic separation (positive selection) (Stem cell kit) and cultured with Mo-DC differentiation medium (Miltenyi Biotec) for seven days. At the end of the 7-day period the induced CD14^neg^CD11c + DCs were classed as immature monocyte-derived dendritic cells (iMoDCs) and cultured in the presence of anti-CD3 antibody.

### Flow cytometry

Staining was performed to determine cell viability (Flexible Viability Dye eFluor™ 780; ThermoFisher) and expression of extracellular/intracellular markers using the following fluorescently-labelled antibodies against human proteins: CD4 (FITC Mab OKT4; ThermoFisher), CD8 (BV711/clone SK1, BioLegend), CD40L (BV605; BioLegend), CD86 (PE/Cy7; BioLegend), NKG2D (PE; BioLegend), CD25 (PE/Cy7; BioLegend**,** CD127 (eFluor450/eBioRDR5; ThermoFisher) within different cell populations. For intracellular staining Brefeldin (3 µg/mL)(Life Technologies) was added to cultures four hours prior to flow cytometry and intracellular staining for Ki67 (Alexa Fluor 488; BioLegend) within CD4+/CD8+ T cells and iMoDC populations and staining for intracellular IFN-γ (PE clone B27; BioLegend). Staining for T regulatory (Treg) cells was performed using anti-Foxp3 (PE conjugate; BioLegend**)** within CD4+/CD127^low^/CD25+ T cells following fixation and permeabilization (Foxp3 transcription factor fixation buffer; ThermoFisher).

### STAT assays

After 72 h in culture, CD3+ T cells were prepared by means of fixation using the BD Phosflow™ Fix buffer I (BD Bioscience) and permeabilised to allow for intracellular staining using BD Phosflow™ Perm Buffer III (BD Bioscience). Cells were then stained with fluorochrome conjugated antibodies detecting CD4 and phospho-STAT1 (PE anti-STAT1, pY701; BD Bioscience), phospho-STAT5 (PE anti-STAT5, pY694, BD Bioscience) and phospho-STAT6 (PE anti-STAT6, pY641, BD Bioscience) and intracellular expression determined within the individual T cell populations by flow cytometry.

### Granzyme B assay

PBMCs were pre-treated with IK14004 for 48 h, then co-cultured with Calcein-AM (BioLegend) stained K562 cells at a 5:1 ratio and incubated for a further 4 h. PBMCs were collected at the end of the culture period and CD8+ T cells assessed for expression of CD107a (APC; BioLegend) by flow cytometry.

### ELISA assays

Supernatants were obtained from PBMC and isolated CD3+ T cells cultures to assess production of IL-2, IL-12p70, IL-10 and IFN-γ (ThermoFisher kits) and IL-12p40 (Biolegend). Isolated CD3+ T cell cultures were also exposed to the Lck inhibitor A-770041 **(**Sigma-Aldrich) (100 nM in 0.1% DMSO v/v) for the duration of experiments in the absence of IK14004. ELISA plates were read at 450 nm using an Infinite F50 (Tecan) absorbance reader and Magellan™ reader control and data analysis software. Flow cytometry data was exported as FCS files from Attune™ NxT software and analysed using FlowJo™ software, from which data were tabulated for export to Microsoft Excel. Graphs and statistical analysis were prepared using Graphpad Prism.

### Statistical analyses

For flow cytometry and ELISA experiments, data from IK14004 and vehicle groups were analysed using parametric statistical procedures. Data within groups to be compared were assumed to be normally distributed and to satisfy the homogeneity of variance criterion. The effect of test compounds (IK14004 or IK14000) was compared with 0.13% water control using one-way or two-way ANOVA with Dunnett’s multiple comparison post-test. Comparison between the effect of Lck inhibitor (A-770041) and 0.1% DMSO control for 7 experimental replicates from two sets of donors was performed using a paired t-test.

### Kinase profiling

Non-cell-based kinase profiling assays were performed by Eurofins (France). All compounds were prepared to 50 × final assay concentration in 100% DMSO. This working stock of the compound was added to assay wells as the first component in the reaction, followed by the remaining components as detailed in the specific assay protocols below. In the standard KinaseProfiler service, there was no pre-incubation step between the compound and the kinase prior to initiation of the reaction. Control wells contained all components of the reaction, except the compound of interest; however, DMSO (at a final concentration of 2%) was included in these wells to control for solvent effects. Blank wells contained all components of the reaction, with a reference inhibitor, staurosporine, replacing the compound of interest in each of the kinase assays. The reference inhibitors for each kinase are indicated in the methods for each kinase assay. These inhibitors abolished kinase activity and established the baseline (0% kinase activity remaining) shown as a dotted line in the graphs. Kinase activities were assessed in the presence of compounds at a concentration range of 0.003 -10 uM and the following kinases were tested: c-Src, Lck, Jak1, Jak2, Tyk2, CaMKIV and HPK1 (MAP4K1). Reactions between IK14004, substrates and MgAcetate/[γ^33^P-ATP] mixes for each respective kinase were stopped by the addition of phosphoric acid and reaction mixtures spotted on P30 filtermats for scintillation according to the detailed methods available from Eurofins. The activities of Lck and c-Src were also assessed in the presence of the unconjugated components of IK14004, i.e., RSKAKNPLYR (IK14000) and the dodecanoic acid moiety [(2)Adod]_4_ (IK00041) and a lipidic peptide containing substituted amino acid residues (IK14DE04).

### Lck binding assays

Lck wild-type (Lck-WT) was prepared in CHO cells**.** The plasmid for the expression of Lck-WT protein was kindly provided by the research laboratory of Professor Katarina Gaus. The plasmid was encoded for mammalian cell expression of the Lck-WT protein with a C-terminal mCherry reporter. A twin-strep tag was incorporated at the N-terminal of the Lck-WT for purification, and a c-myc epitope tag was placed at the C-terminal, after mCherry for detection of protein expression. A midiprep DNA preparation of the plasmid was performed using the Macherey–Nagel Midi kit as per manufacturer’s instructions. For transient transfections plasmid DNA was transfected into CHO-S cells using 2 μg DNA mL^−1^ cells at a concentration of 3 million cells mL^−1^. DNA was complexed with polyethylenimine-Pro (PolyPlus) in Opti-Pro serum free medium (Life Technologies) at a DNA (μg) to PEI (μL) ratio of 1:4 (w:v) for 15 min prior to transfecting suspension adapted CHO cells. The transfected cells were cultured in chemically defined CHO medium (CD-CHO; Life Technologies) at 37 °C, 7.5% CO_2_, 70% humidity with shaking at 130 rpm for 6 h, before feeding with 7.5% CD CHO Efficient Feed A (Life Technologies), 7.5% CD-CHO Efficient Feed B (Life Technologies), and 0.4% anti-clumping agent (Gibco) and continuing the culture at 32 °C, 7.5% CO_2_, 70% humidity with shaking at 130 rpm for 2 days. Following transfection, the cells were pelleted by centrifugation at 5250*g* for 30 min. The cells were briefly sonicated with 2 pulses of 30 secs on/off using the Vibra-Cell™ VC 505 sonicator (Sonics). Cells were centrifuged at 5250*g* for 10 min and supernatant was collected and filtered through a 0.22 μm membrane (Sartorius). The Lck-WT protein was purified from the clarified supernatant using a 5 mL Strep-Trap column (GE). Protein was eluted using desthiobiotin.

The peptides, IK14004 and IK14000, were reconstituted into PBS at 1 mg/mL. Each peptide stock was diluted in PBS to 10 µg/mL and 100 µL added to wells of a maxisorp plate (Nunc). Wells were coated at 4 °C for 20 h. Triplicate wells were used for testing the binding of different concentrations of the purified Lck-WT protein. Following coating, the wells were blocked with 200 µL of 2% Milk-PBST (0.05% Tween 20 in PBS) for 1 h. The blocker was then decanted and 100 µL of Lck-WT diluted in PBS was added and incubated at room temperature for 2 h. The wells were then washed 4 times with PBST (200 µL per well/wash) and 100 µL of HRP anti myc (Miltenyi Biotech) diluted 1/5000 in block solution was added for 1 h at room temperature. The wells were then washed 4xPBST and 100 µL of TMB (Sigma) was added per well for 10 min. 100 µL of 2 M sulphuric acid was added to stop the reaction and the absorbance values recorded at a wavelength of 450 nm using SpectraMax (Molecular Devices).

## Supplementary Information

Supplementary Figures.
