# Supplementary material for: A novel lipidic peptide with potential to promote balanced effector-regulatory T cell responses
Source: Sci Rep. 2022 Jul 1;12:11185. doi: 10.1038/s41598-022-15455-5 (PMC9249808; doi:10.1038/s41598-022-15455-5)
Supplement: Supplementary file 1 — Supplementary Figures. [file 41598_2022_15455_MOESM1_ESM.pdf]

Supplementary information online
flow cytometry data

Figure S1 - Refers to manuscript **Fig. 1a:** Ki67 MFI in CD4+ (CD3 72 hrs)

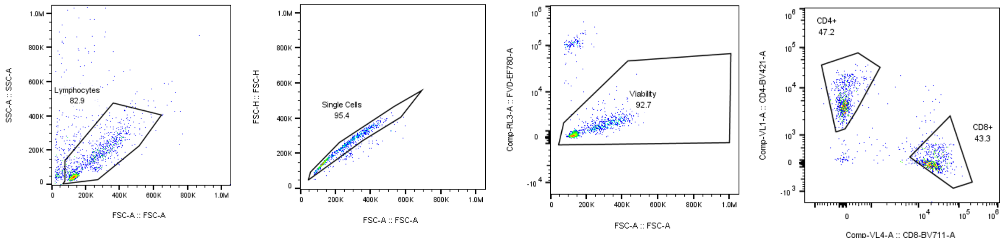

FMO vs IK14004 1.25 µM

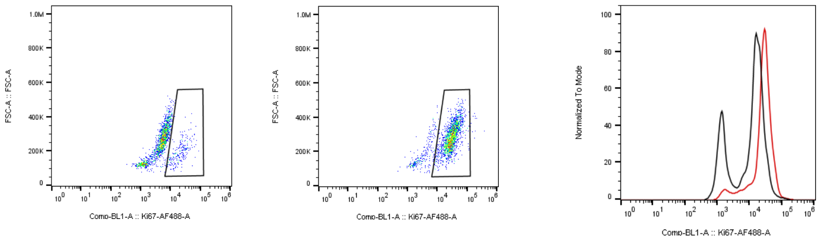

Black line = Vehicle
Red line = IK14004 (1.25 µM)

Figure S2 - Refers to manuscript **Fig. 1b:** CD25 MFI in CD4+ (CD3 72 hrs)

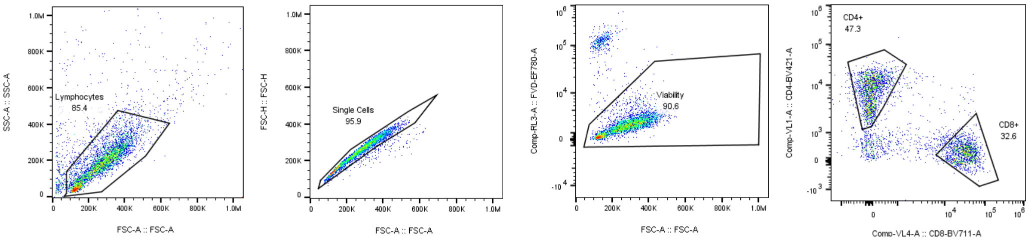

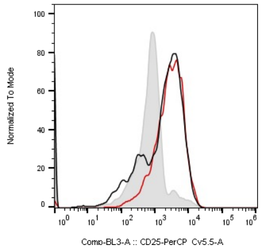

Grey area = FMO
Black line = Vehicle
Red line = IK14004 (1.25 µM)

Figure S3 - Refers to manuscript **Fig. 1c:** Ki67 MFI in CD8+ (CD3 72 hrs)

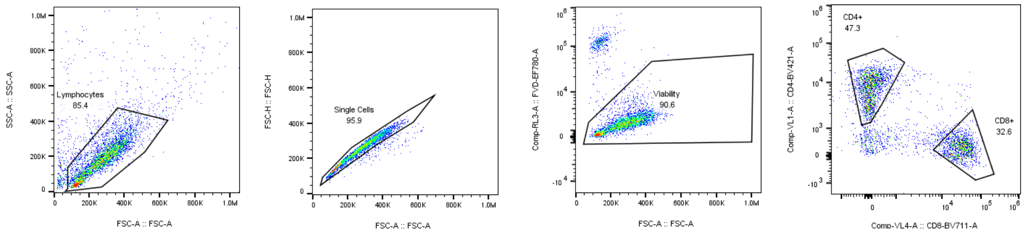

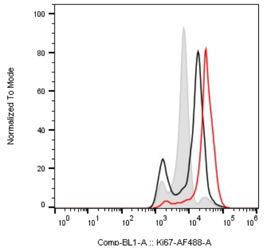

Grey area = FMO
Black line = Vehicle
Red line = IK14004 (1.25 µM)

Figure S4- Refers to manuscript **Fig. 1d:** CD25 MFI in CD8+ (CD3 72 hrs)

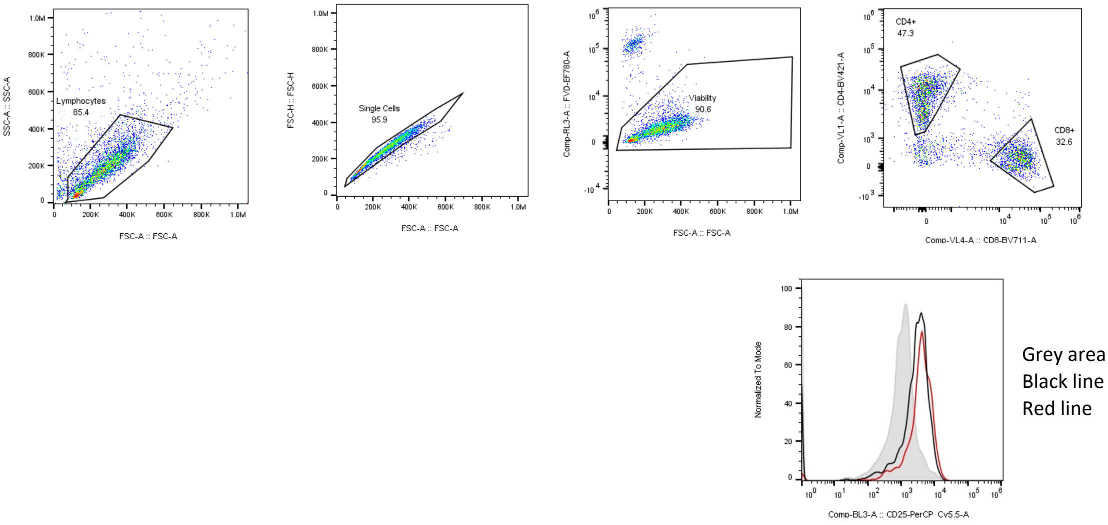

Grey area = FMO
Black line = Vehicle
Red line = IK14004 (1.25 µM)

Figure S5 - Refers to manuscript **Fig. 1e:** CD25 MFI in CD4+ (CD3 72 hrs)

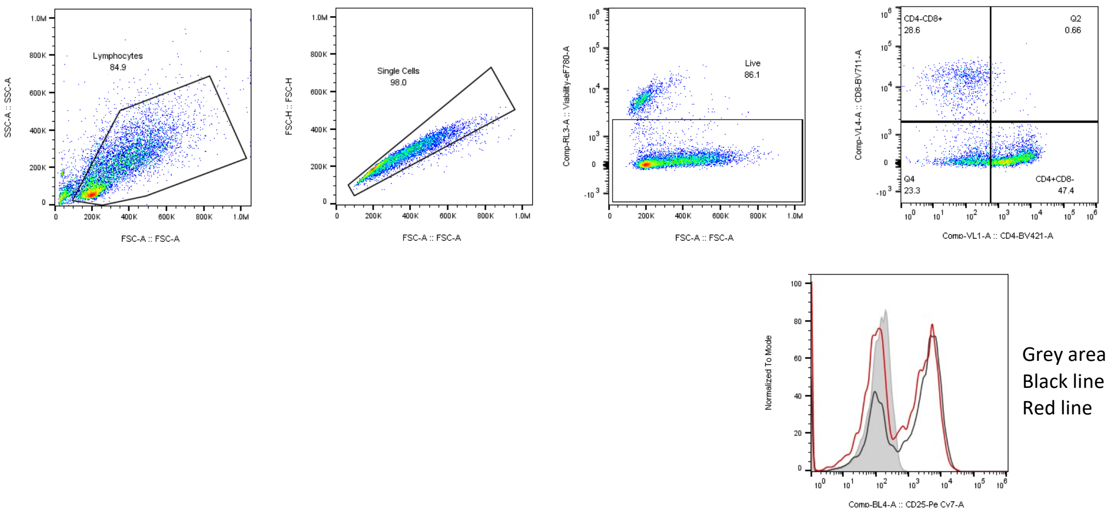

Grey area = FMO
Black line = Vehicle
Red line = IK14004 (1.25 µM)

Figure S6 - Refers to manuscript **Fig. 1h:** Ki67 MFI in CD4+ (PBMC 72 hrs)

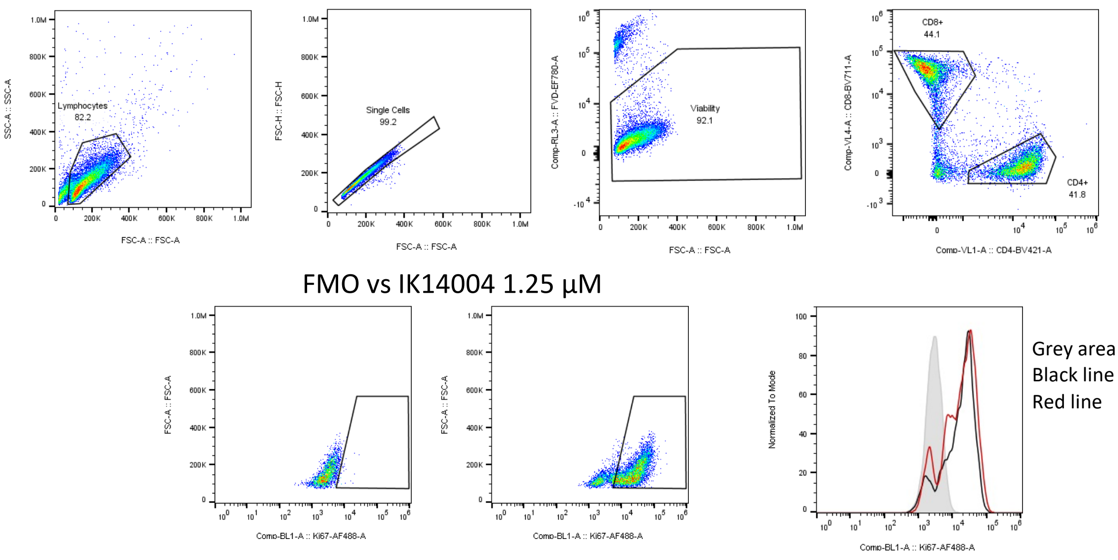

Grey area = FMO
Black line = Vehicle
Red line = IK14004 (1.25 µM)

Figure S7 - Refers to manuscript **Fig. 1i:** CD25 MFI in CD4+ (PBMC 72 hrs)

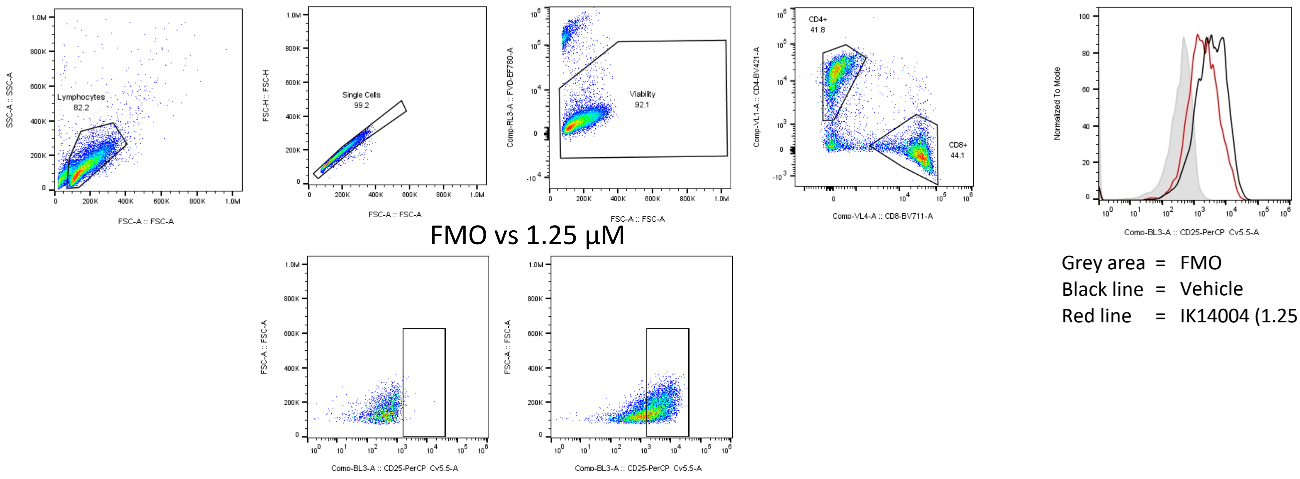

Grey area = FMO
Black line = Vehicle
Red line = IK14004 (1.25 µM)

Figure S8 - Refers to manuscript **Fig. 1j:** Ki67 MFI in CD8+ (PBMC 72 hrs)

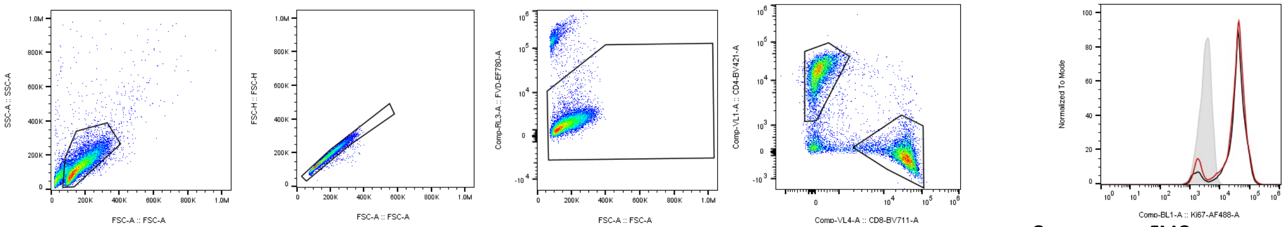

Grey area = FMO
Black line = Vehicle
Red line = IK14004 (1.25 µM)

Figure S9 - Refers to manuscript **Fig. 1k:** CD25 MFI in CD8+ (PBMC 72 hrs)

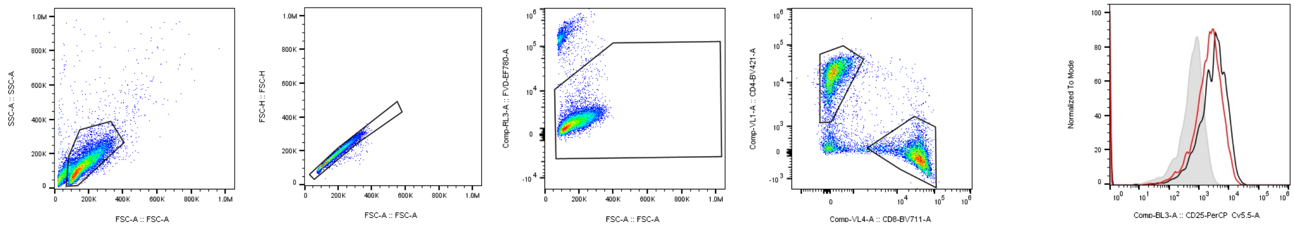

Grey area = FMO
Black line = Vehicle
Red line = IK14004 (1.25 µM)

Figure S10 - Refers to manuscript **Fig. 2a:** CD40L MFI in CD4+ (CD3 72 hrs)

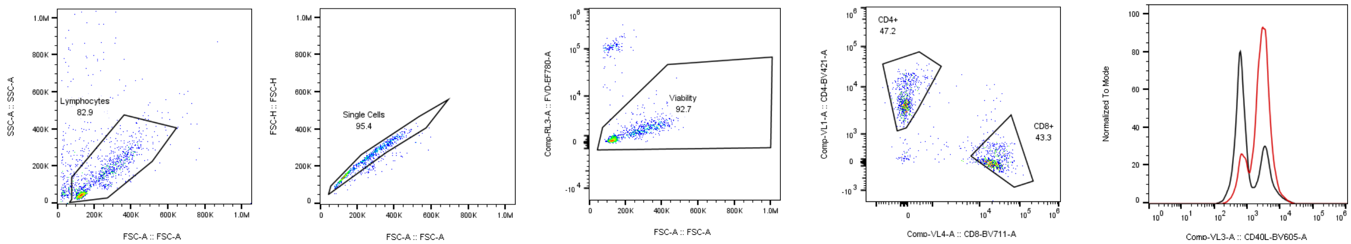

Black line = Vehicle
Red line = IK14004 (1.25 µM)

Figure S11 - Refers to manuscript **Fig. 2b:** CD40L MFI in CD4+ (PBMC 72 hrs)

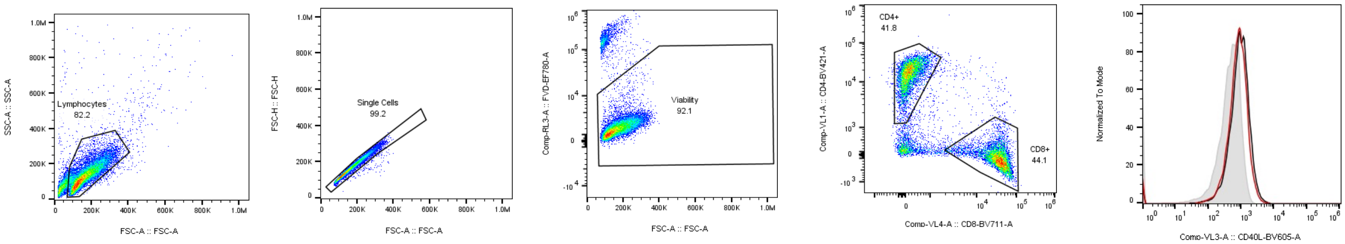

Grey area = FMO
Black line = Vehicle
Red line = IK14004 (1.25 µM)

Figure S12 - Refers to manuscript **Fig. 2c:** CD40L MFI in CD8+ (CD3 72hrs)

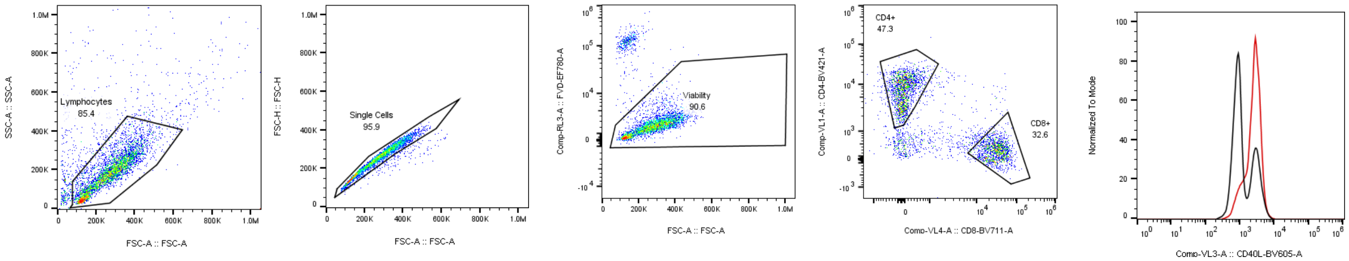

Black line = Vehicle
Red line = IK14004 (1.25 µM)

Figure S13 - Refers to manuscript **Fig. 2d:** CD40L MFI in CD8+ (PBMC 72 hrs)

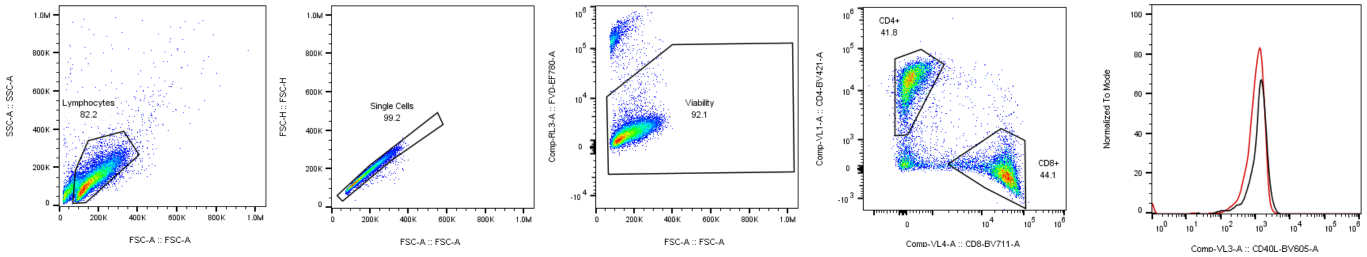

Black line = Vehicle
Red line = IK14004 (1.25 µM)

Figure S14 - Refers to manuscript **Fig. 2e:** Viability iMoDC (72 hrs)

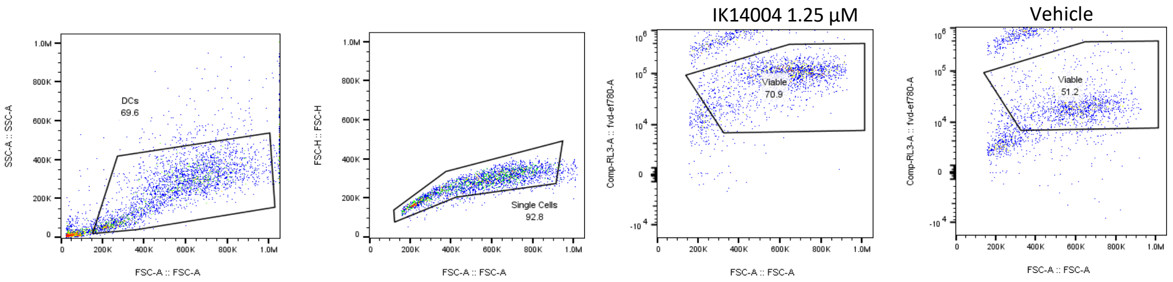

Figure S15 - Refers to manuscript **Fig. 2f:** CD86 MFI in iMoDC (72 hrs)

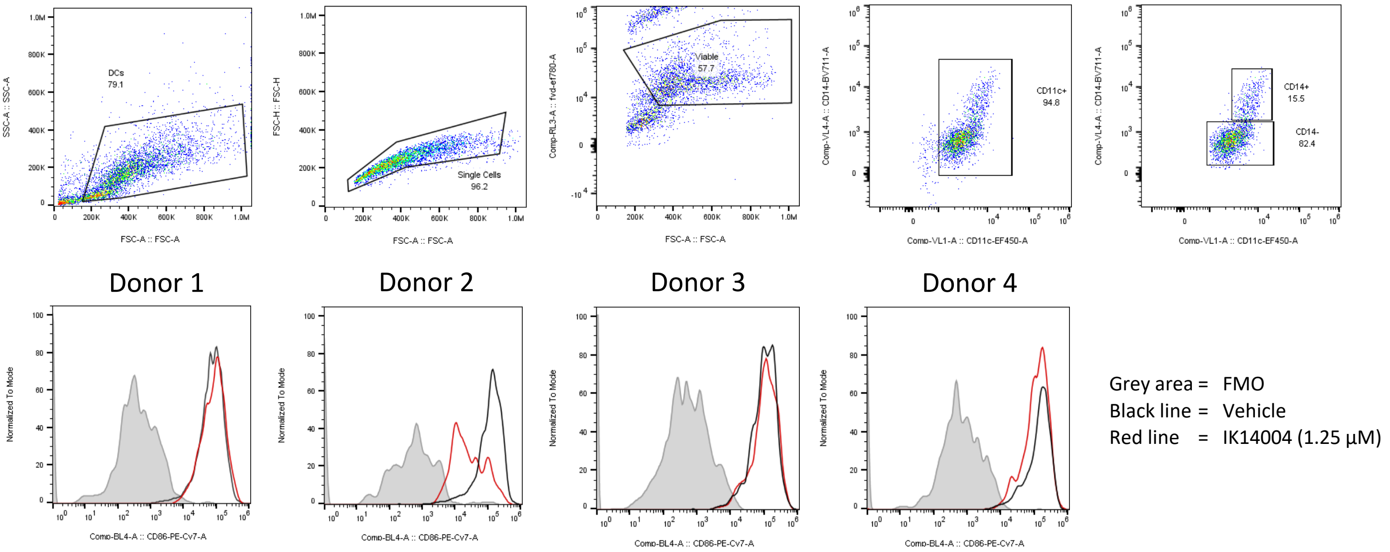

Figure S16 - Refers to manuscript **Fig. 2g:** CD25 MFI in iMoDC (72 hrs)

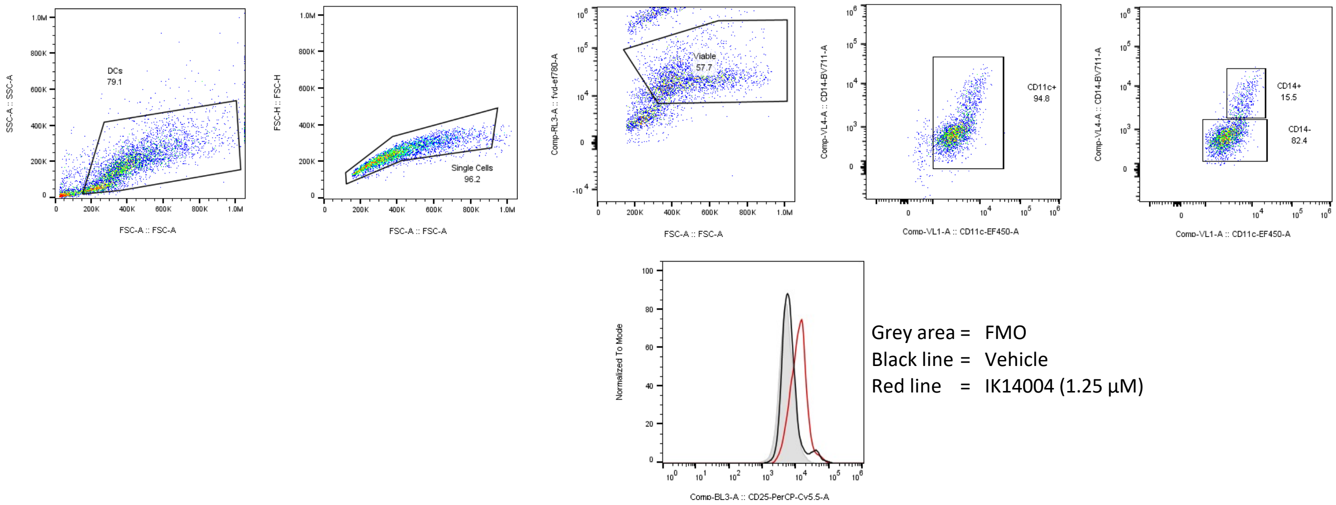

Figure S17 - Refers to manuscript **Fig. 2j:** Viability iMoDC (CD14+ versus CD14-)

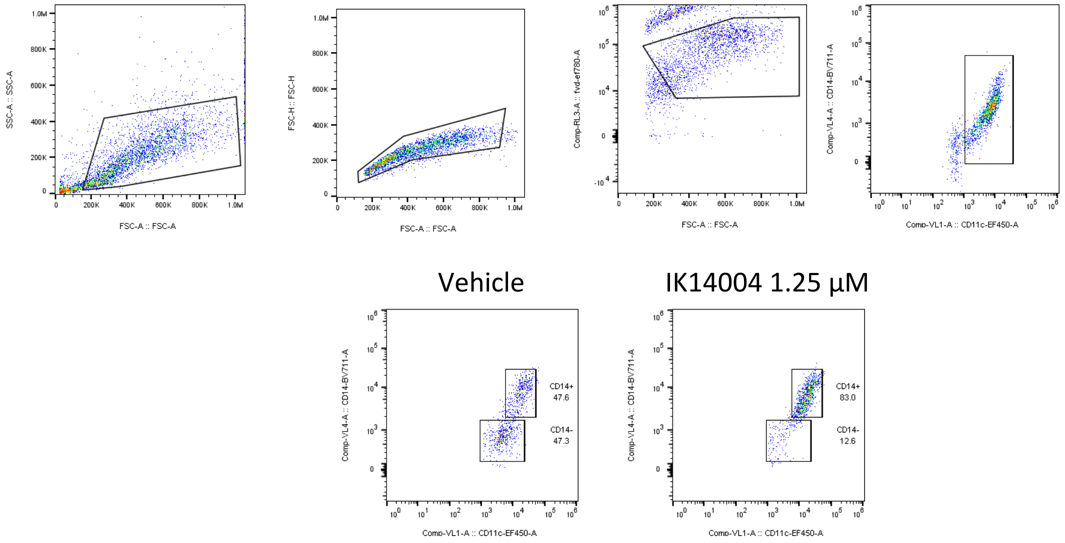

Figure S18 - Refers to manuscript **Fig. 3a**: CD28 % in CD8+ (PBMC 72 hrs)

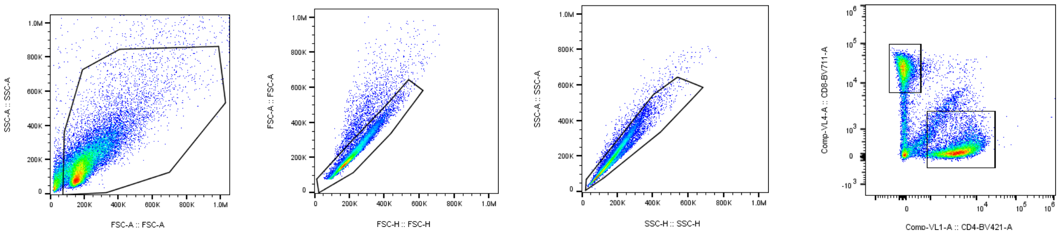

Gating shows CD28- and CD28+

IgG1 Isotype control   IK14004 1.25 µM

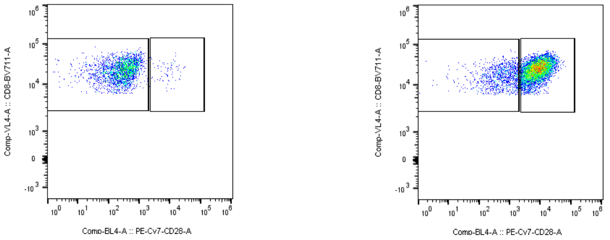

Figure S19 - Refers to manuscript **Fig. 3b**: Ki67 % in CD8+ CD28+ (PBMC 72 hrs)

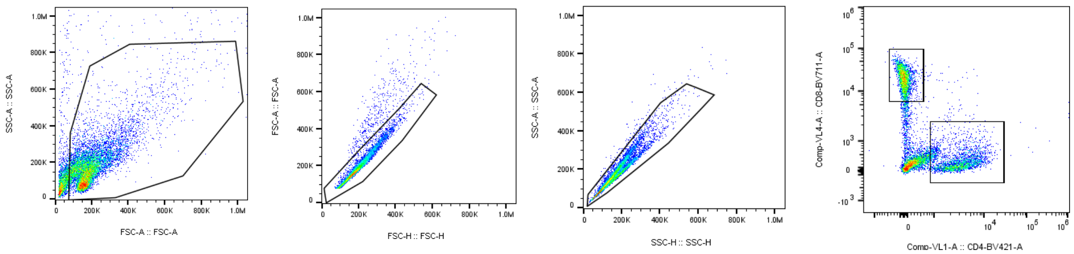

CD8+CD28+ Vehicle   CD8+CD28+ IK14004 1.25 µM

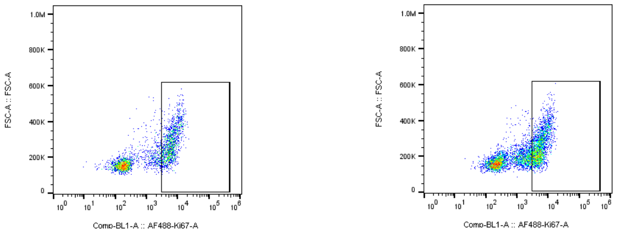

Figure S20 - Refers to manuscript **Fig. 3c**: CD28 % in CD4+ (PBMC 72 hrs)

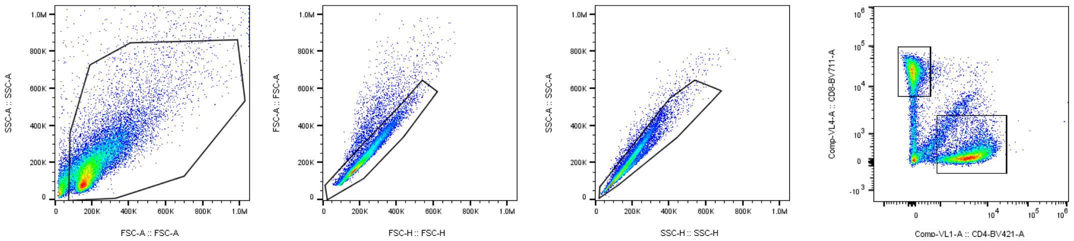

Gating shows CD28- and CD28+

IgG1 Isotype control   IK14004 1.25 µM

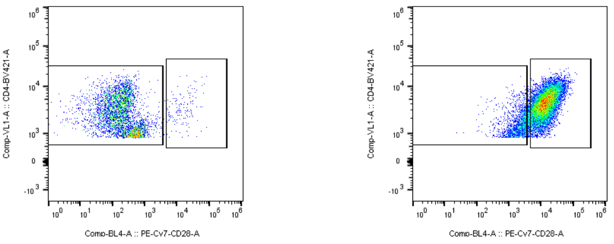

Figure S21 - Refers to manuscript **Fig. 3d**: Ki67 % in CD4+ CD28+ (PBMC 72 hrs)

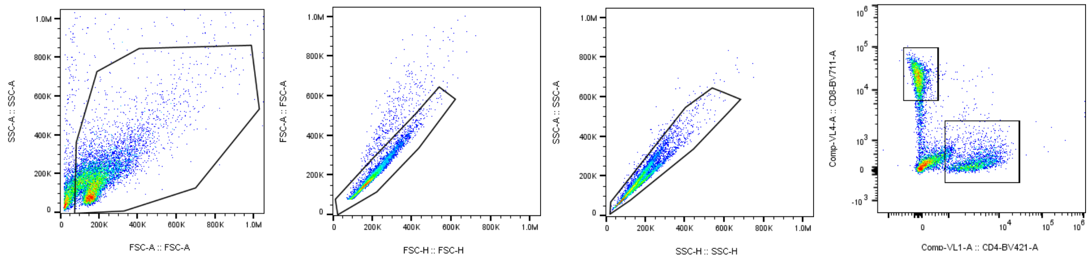

CD4+CD28+ Vehicle   CD4+CD28+ IK14004 1.25 µM

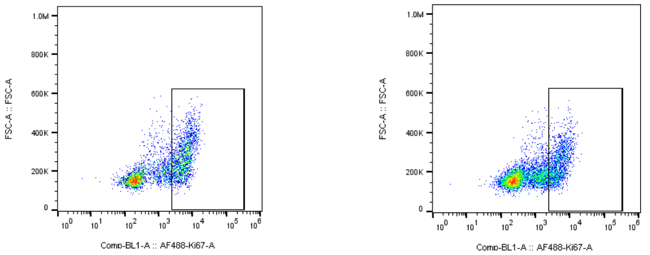

Figure S22 - Refers to manuscript **Fig. 3e**: NKG2D MFI in CD8+ (PBMC 72 hrs)

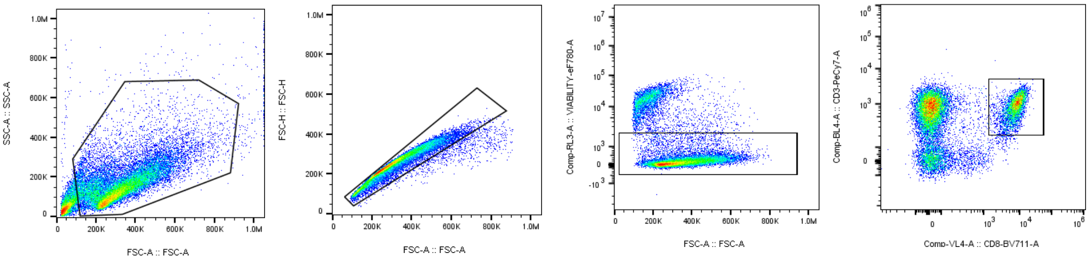

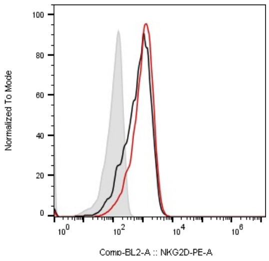

Grey area = FMO
Black line = Vehicle
Red line = IK14004 (1.25 µM)

Figure S23 - Refers to manuscript **Fig. 3f**: CD107a % in CD8+ (PBMC:K562 co-culture 48 hrs)

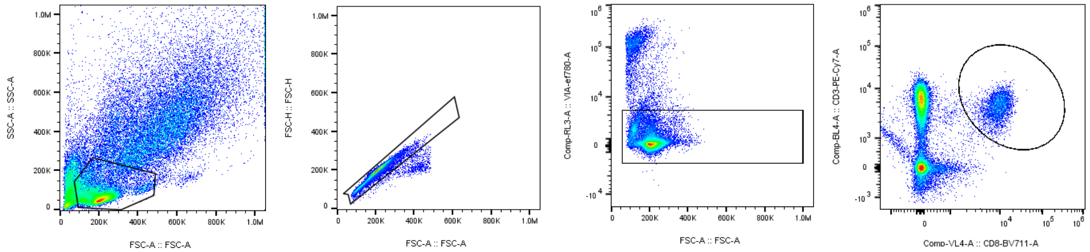

IgG1 Isotype vs IK14004 1.25 µM

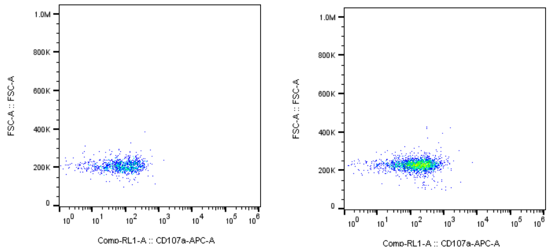

Figure S24 - Refers to manuscript **Fig. 3g**: pSTAT5 MFI in CD8+ (CD3 72 hrs)

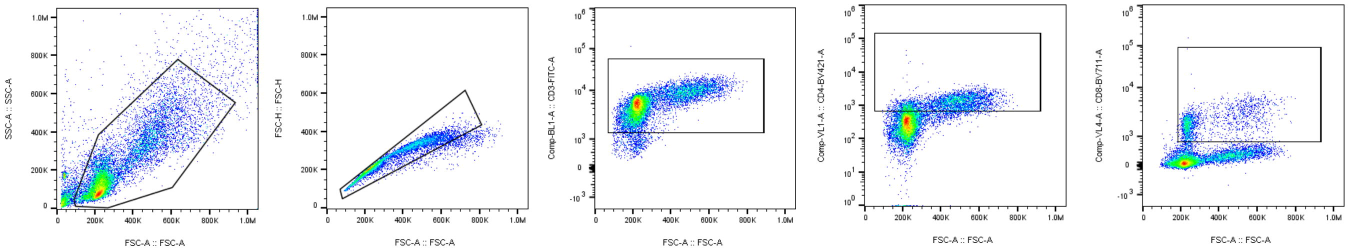

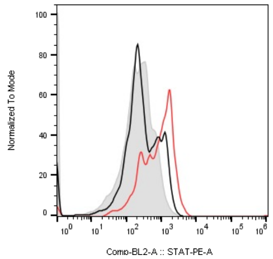

Grey area = FMO
Black line = Vehicle
Red line = IK14004 (1.25 µM)

Figure S25 - Refers to manuscript **Fig. 3h**: pSTAT5 % in CD8+ (CD3 72hrs)

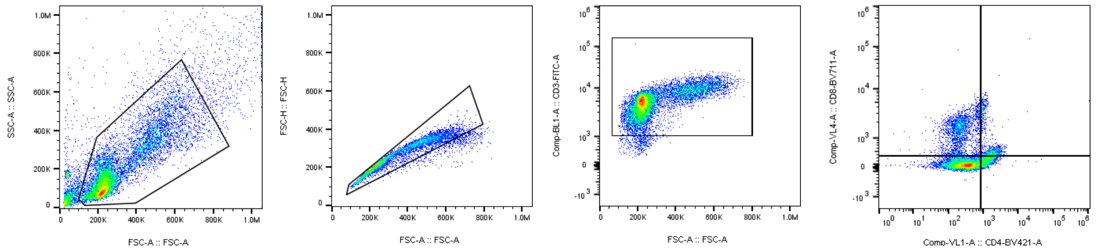

FMO control

IK14004 1.25 µM

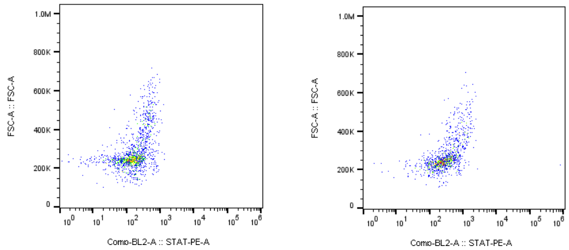

Figure S26 - Refers to manuscript **Fig. 3i**: pSTAT5 MFI in CD4+ (CD3 72hrs)

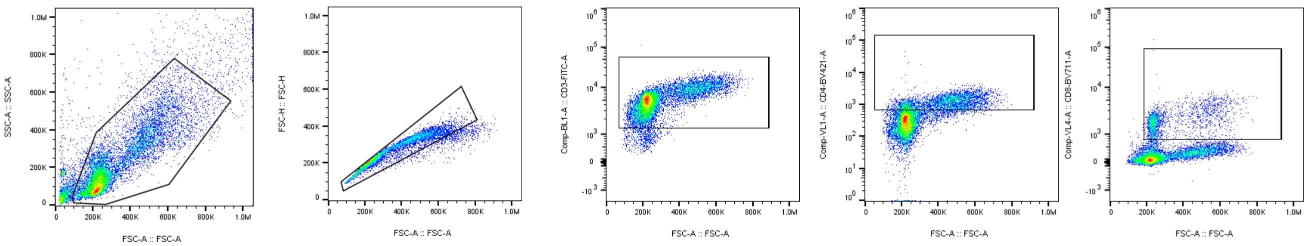

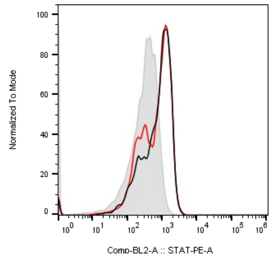

Grey area = FMO
Black line = Vehicle
Red line = IK14004 (1.25 µM)

Figure S27 - Refers to manuscript **Fig. 3j**: pSTAT5 % in CD4+ (CD3 72hrs)

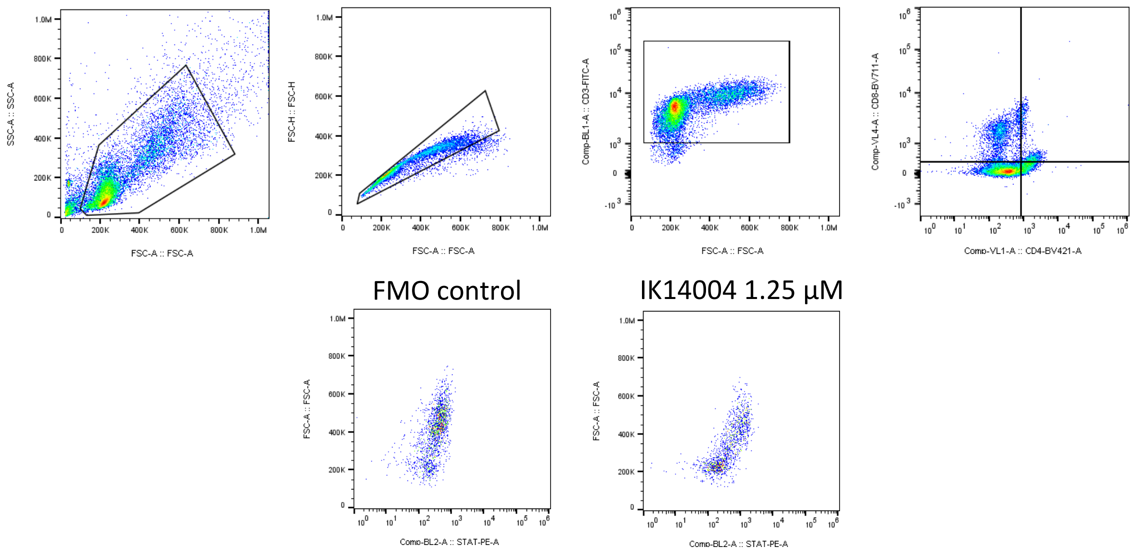

Figure S28 - Refers to manuscript **Fig. 5a**: intracellular IFN-γ % in CD4+ (PBMC 24 hrs)

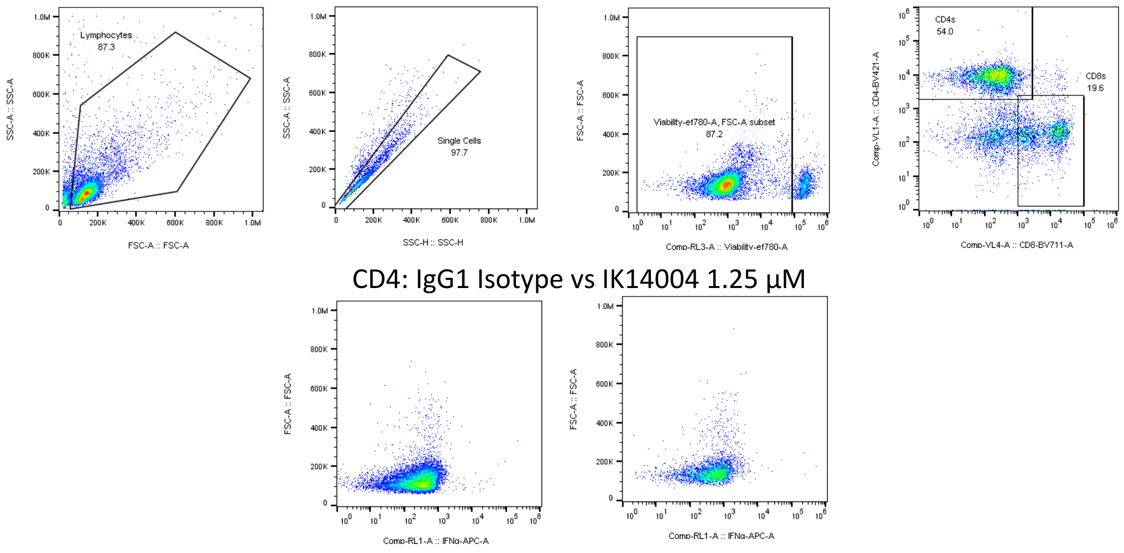

Figure S29 - Refers to manuscript **Fig. 5b**: intracellular IFN-γ % in CD8+ (PBMC 24 hrs)

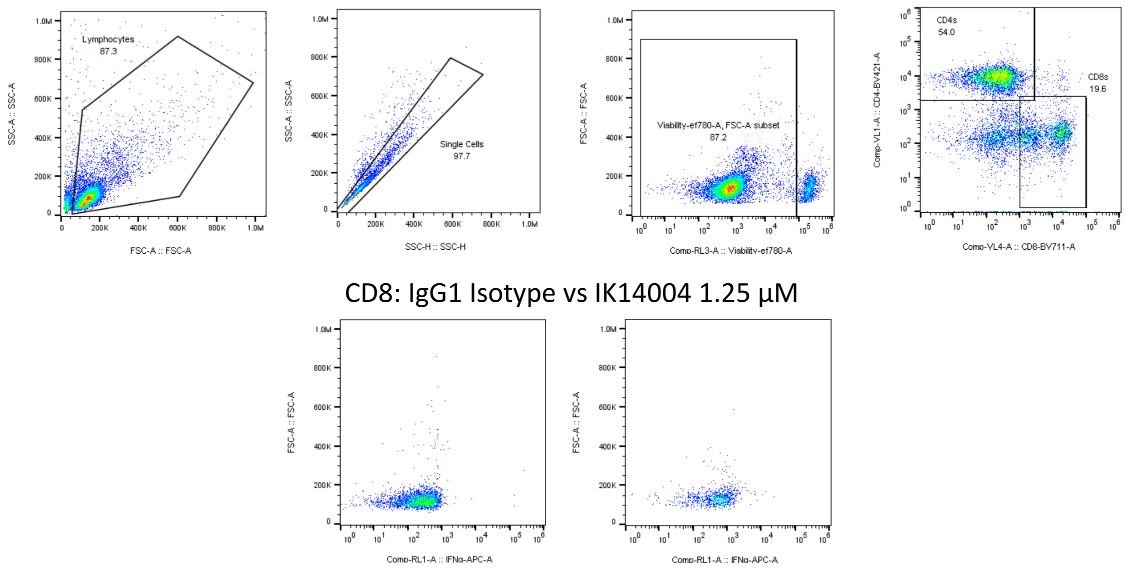

Figure S30 - Refers to manuscript **Fig. 5c**: intracellular IFN-γ MFI in CD4+ (PBMC 24 hrs)

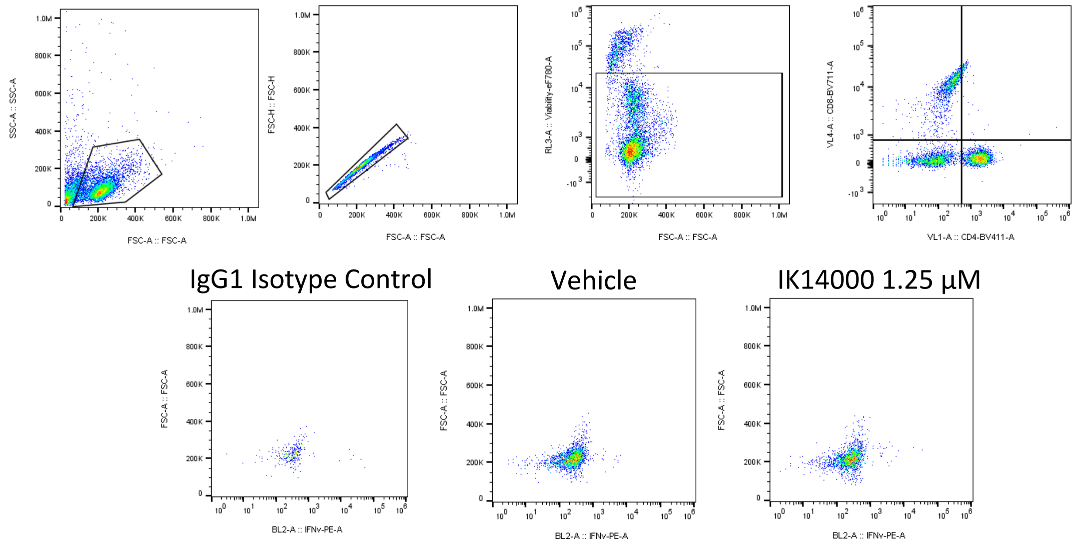

Figure S31 - Refers to manuscript **Fig. 5d**: intracellular IFN-γ MFI in CD8+ (PBMC 24 hrs)

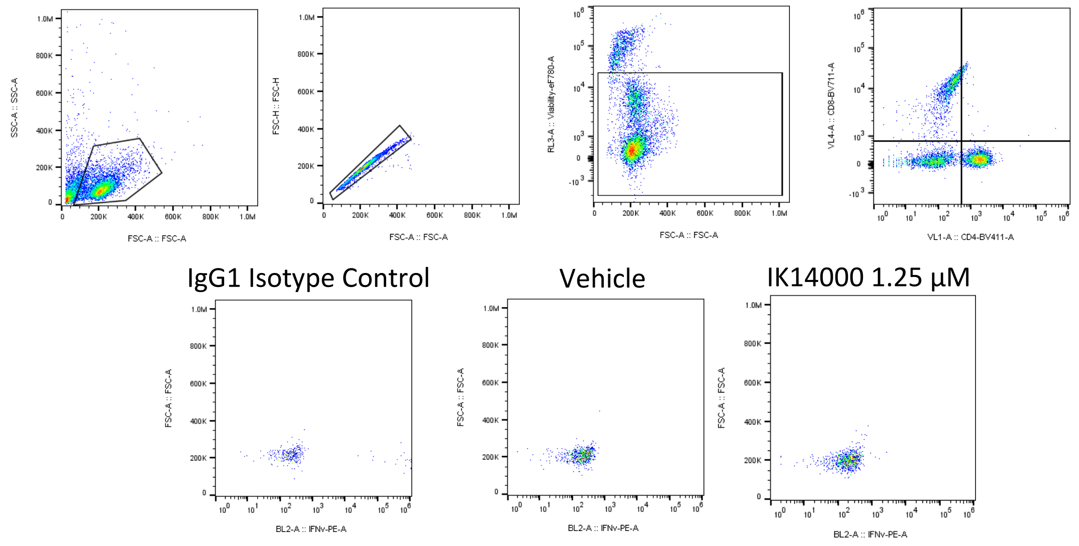

Figure S32 - Refers to manuscript **Fig. 5h**: CD25 % within CD4+ (PBMC 72 hrs)

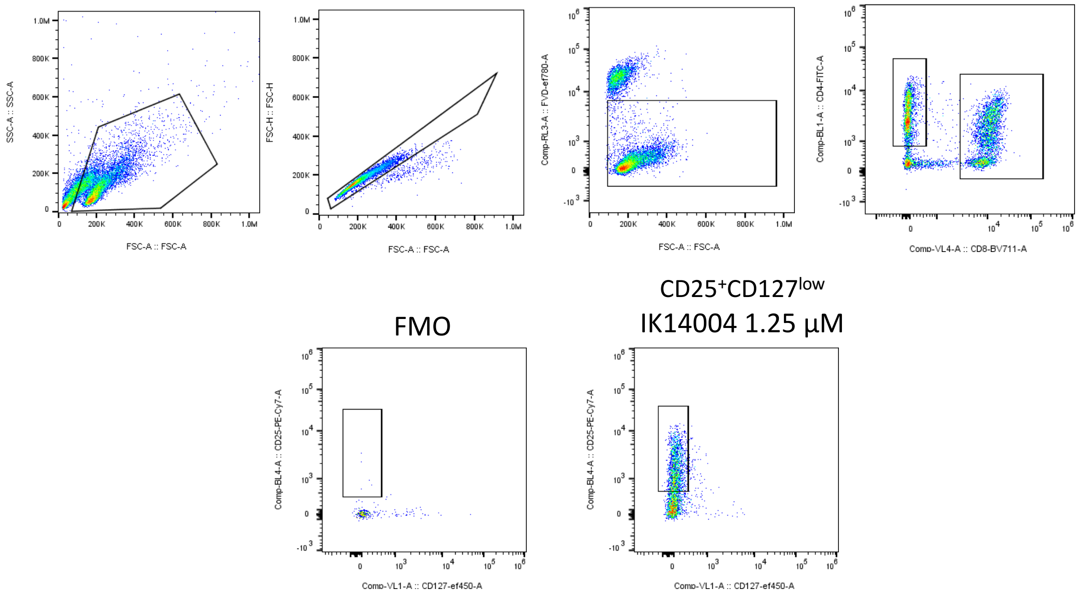

Figure S33 - Refers to manuscript **Fig. 5i**: Foxp3 % in CD4+ CD127^{low} (PBMC 72 hrs)

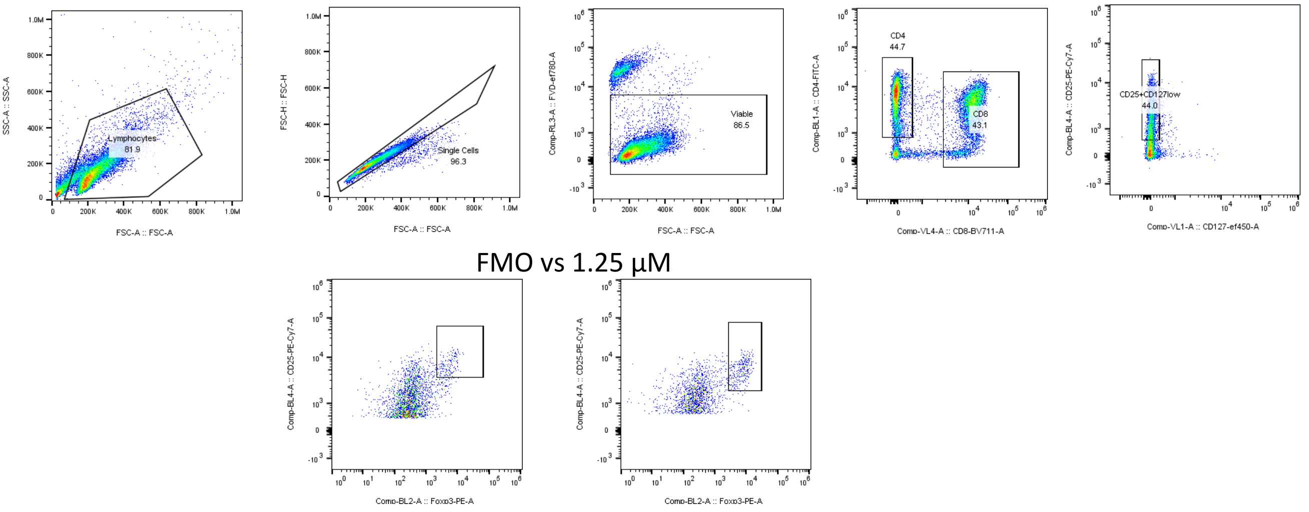

Figure S34 - Refers to manuscript **Fig. 5j**: CD4/Treg ratio (PBMC 72 hrs)

Identification of CD4^{+}CD25^{+}CD127^{lo}FOXP3^{+} Tregs

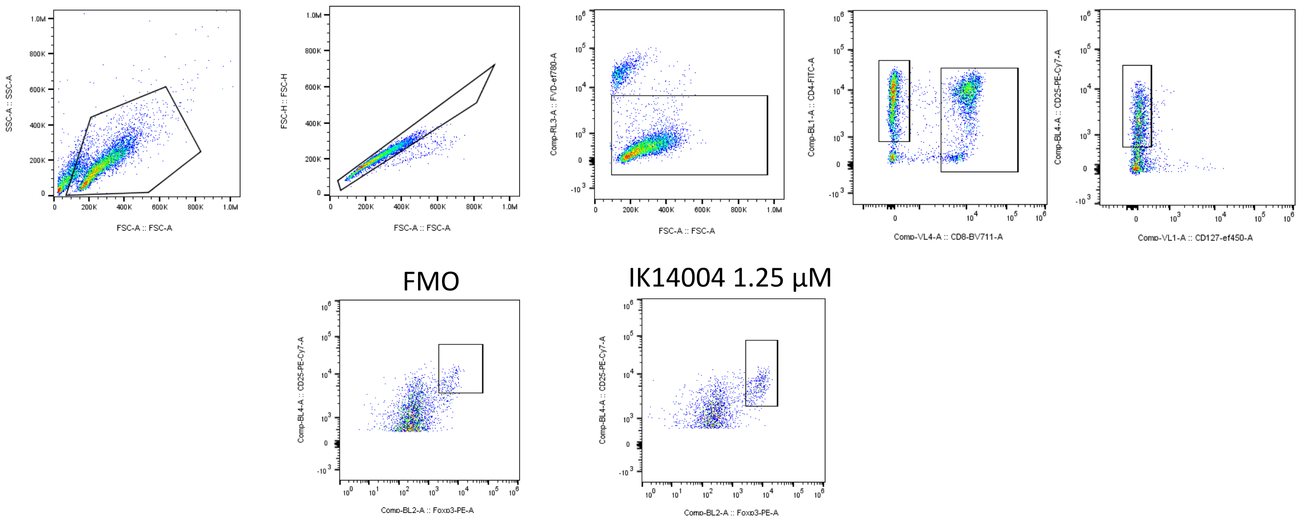

Figure S35 - Refers to manuscript **Fig. 5k**: Foxp3 MFI (PBMC 72 hrs)

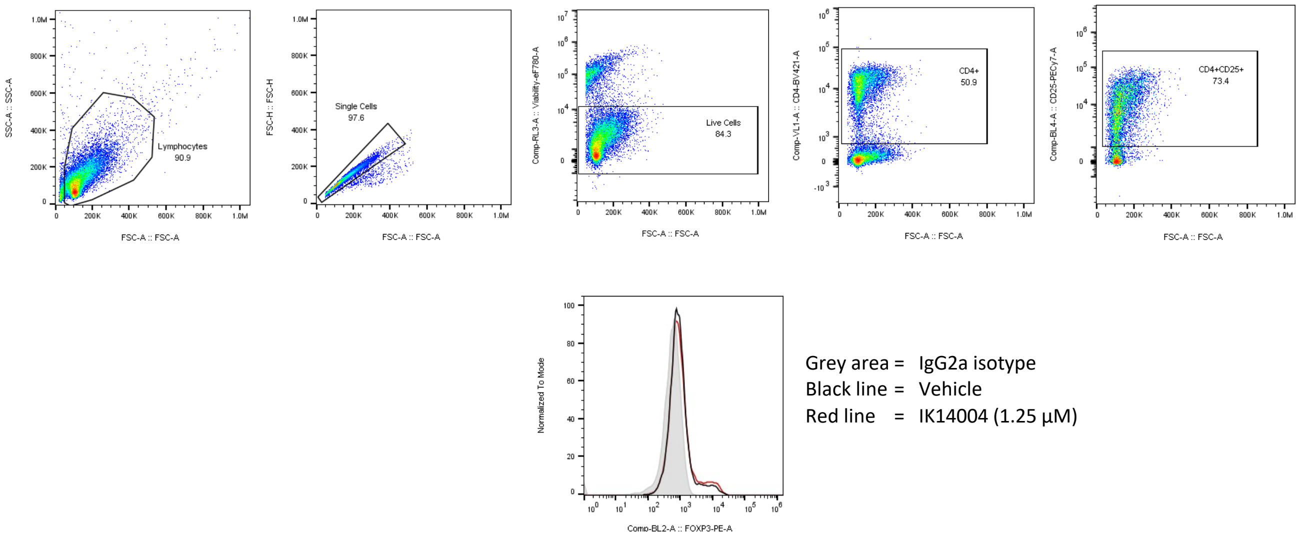

Grey area = IgG2a isotype
Black line = Vehicle
Red line = IK14004 (1.25 μM)

Figure S36 - Refers to manuscript **Fig. 6g**: pSTAT1 MFI in CD4+ (CD3 72 hrs)

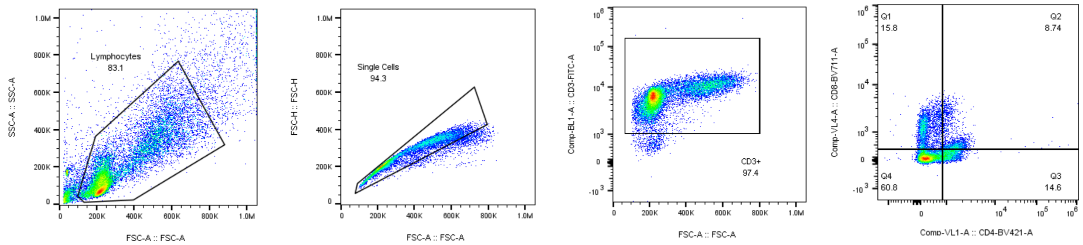

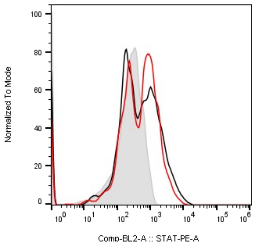

Grey area = IgG2a isotype
Black line = Vehicle
Red line = IK14004 (1.25 µM)

Figure S37 - Refers to manuscript **Fig. 6h**: pSTAT6 MFI in CD4+ (CD3 72 hrs)

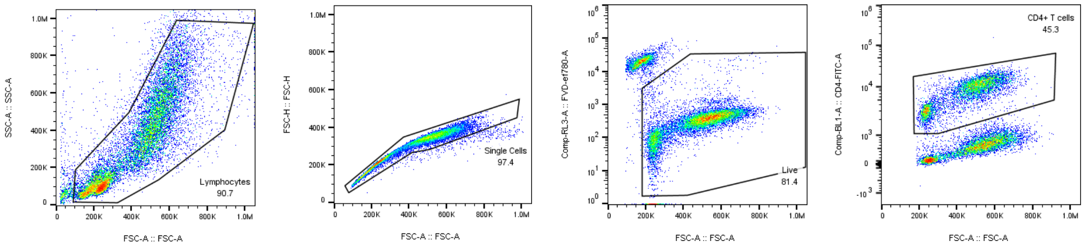

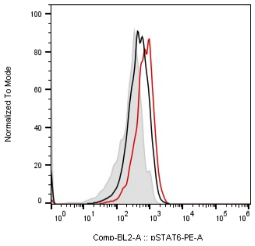

Grey area = IgG2a isotype
Black line = Vehicle
Red line = IK14004 (1.25 µM)
